# Red-Light-Induced PET-RAFT Polymerization to Afford (Meth)acrylamide-Based Poly(*N*‑oxide) and Other Hydrophilic Polymers Featuring Neutral, Cationic, and Zwitterionic Groups as Solubilizing Side Chains

**DOI:** 10.1021/acs.macromol.5c03186

**Published:** 2026-02-13

**Authors:** Van-Sieu Luc, Kien-Sam Banh, Thach-Thao T. Nguyen, Min-Hsuan Hsieh, Tung-Kung Wu, Vitalijus Karabanovas, Ricardas Rotomskis, Simona Steponkiene, Yaw-Kuen Li, Ying-Nien Chou, I-Chi Lee, Chia-Chih Chang

**Affiliations:** † Department of Applied Chemistry, 34914 National Yang Ming Chiao Tung University, Hsinchu 300093, Taiwan; ‡ Institute of Chemistry, Academia Sinica, Taipei 11529, Taiwan; § Sustainable Chemical Science and Technology (SCST), Taiwan International Graduate Program (TIGP), Academia Sinica, Taipei 11529, Taiwan; ∥ Department of Biomedical Engineering and Environmental Sciences, 34881 National Tsing Hua University, Hsinchu 300044 Taiwan; ⊥ Department of Biological Science and Technology, 498907 National Yang Ming Chiao Tung University, Hsinchu 300093, Taiwan; # Biomedical Physics Laboratory, 277613 National Cancer Institute, Vilnius LT-08406, Lithuania; ∇ Department of Chemical Engineering, National Chung Cheng University, Minhsiung, Chiayi 62102, Taiwan; ○ Center for Emergent Functional Matter Science, National Yang Ming Chiao Tung University, Hsinchu 300093, Taiwan

## Abstract

Amine-oxide-containing polymers, poly­(*N*-oxide), have emerged as an alternative to poly­(ethylene glycol) (PEG) for imparting biofouling resistance and enabling drug delivery applications. Poly­(*N*-oxide) can be obtained by thermally initiated controlled free radical polymerization and postpolymerization modification of the corresponding tertiary amine-containing polymers. However, unexpected side reactions between (meth)­acrylamide-based *N*-oxide monomers and chain transfer agents commonly used in thermally initiated reversible addition–fragmentation chain transfer (RAFT) polymerization present challenges for achieving controlled polymerization. Herein, this study exploits visible-light-induced photoelectron transfer (PET)-RAFT polymerization of *N*-oxide-containing (meth)­acrylamides by using two types of zinc­(II) porphyrin derivatives. This approach affords well-defined (meth)­acrylamide- and methacrylate-based *N*-oxide polymers with narrow molecular weight distributions even at a catalyst loading of less than 100 ppm. Successful one-pot chain- extension experiments confirm the good end-group fidelity. The optimized reaction condition can afford well-defined polymers with *M*_n_ up to 126 kDa. This method enables polymerization under complete oxygen tolerance, with excellent temporal control and the ability to conduct polymerization from low volume to large scale without diminishing the control over polymerization. In addition, such a method is also applicable to other hydrophilic methacrylate monomers featuring neutral oligo­(ethylene glycol), zwitterionic sulfobetaine, and phosphocholine, as well as cationic quaternary ammonium groups as side chains.

## Graphical abstract

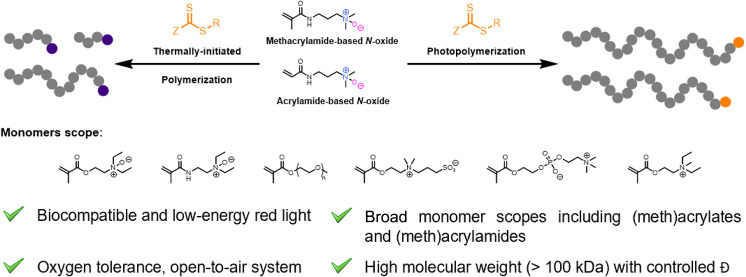

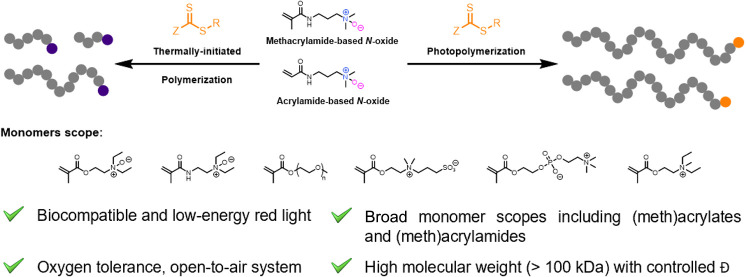

## 1 Introduction

Reversible deactivation radical polymerization (RDRP) techniques, including atom transfer radical polymerization (ATRP) and reversible addition–fragmentation chain transfer (RAFT) polymerization, have transformed the landscape of polymeric materials, enabling access to unprecedented materials through controlled polymerization of functional monomers.^1^ Photopolymerization has been widely implemented in various fields owing to its numerous advantages, such as low energy requirements, ease of operation, mild conditions, and excellent temporal controllability. The growing interest in photoinduced RDRP processes has led to the discovery of many reaction systems over the past two decades.^2–4^ Matyjaszewski and coworkers reported photoinduced ATRP under ultraviolet (392 nm) and blue light (450 nm) irradiation, enabling controlled polymerization of methyl methacrylate (MMA) and methyl acrylate (MA) monomers.^5^ Hawker and coworkers developed another photoinduced ATRP system using ppm levels of *fac*-[Ir­(ppy)_3_] as the photocatalyst (PC) under irradiation with a 50 W fluorescent lamp. The polymerization of methacrylates can be reversibly activated and deactivated through light on/off cycles while maintaining excellent control over molecular weight and molecular weight distribution (Đ).^6^ Boyer and coworkers extended the use of *fac*-[Ir­(ppy)_3_] with LED light (4.8 W) irradiation to achieve photoinduced RDRP in the presence of a chain transfer agent (CTA), namely, photoinduced electron/energy transfer reversible addition–fragmentation chain transfer (PET-RAFT) polymerization in 2014.^7^ A large range of monomers, including (meth)­acrylates, (meth)­acrylamides, styrene, vinyl acetate, vinyl phosphonate, etc., have been polymerized by using ultralow concentrations of PC and a low-energy visible LED (1–4.8 W, λ_max_= 435 nm). The scope of PCs has been expanded to enable PET-RAFT polymerization of various monomers across a wide range of wavelengths, which can be further classified into 3 groups: organometallic PCs (i.e., Ir­(ppy)_3_,^7^ Ru­(bpy)_3_Cl_2_,^8^ chlorophyll a^9^); organic PCs (i.e., Eosin Y,^10^ Rose Bengal,^11^ Phenothiazines,^12^ Cyanoarenes^13^); and inorganic PCs (i.e., Ag_3_PO_4_,^14^ Bi_2_O_3_,^15^ CdSe QDs,^16^ BiOCl nanosheets^17^). However, most of these PCs are activated under short irradiation wavelengths (UV, blue, and green light), thus limiting their application in biological systems.

The use of red light for polymer synthesis has emerged as a popular light source due to its high biocompatibility, tissue penetrability, low scattering, and minimal side reactions.^9,18–20^ Boyer and coworkers pioneered the use of zinc­(II) *meso*-tetraphenylporphyrin (ZnTPP) as a versatile photocatalyst that can be activated under a wide range of irradiation wavelengths (435–655 nm). The controlled polymerization of styrene, (meth)­acrylates, and (meth)­acrylamides could be performed at a low PC loading (50 ppm) with excellent oxygen tolerance under various irradiation wavelengths. The polymerization rate is highly dependent on the choice of light sources.^21^ ZnTPP in DMSO proves to be a versatile, controlled PET-RAFT system. Boyer and coworkers further developed a water-soluble zinc porphyrin derivative, zinc­(II) *meso*-tetra­(4-sulfonatophenyl)­porphyrin (ZnTPS_4_), which enabled PET-RAFT polymerization in aqueous media without the need for deoxygenation.^22^ These platforms open an avenue for the synthesis of DNA–, RNA–, and protein–polymer conjugates, as well as the facile grafting of polymers onto various substrates.^23–29^

Recently, a class of novel hydrophilic polymers based on amine-oxide (*N*-oxide) was reported as the next generation of nonfouling materials. In 2019, Jiang and coworkers reported poly­(trimethylamine *N*-oxide) (pTMAO) with ultralow fouling properties and minimal immunogenicity compared to the commonly used PEG.^30^ Trimethylamine-*N*-oxide (TMAO), a small organic molecule found in many organisms, is known as an important osmoprotectant that overcomes biochemical stress experienced by proteins due to the high intracellular concentration of urea.^31^ Unlike conventional zwitterionic polymers (ZPs) that have at least one carbon spacer between the positively and negatively charged groups, TMAO has a negatively charged oxygen atom directly attached to the positively charged quaternary ammonium group. Such a minimal distance increases the dipole moment and the hydration capacity and thus boosts the fouling resistance performance.^32^ Poly­(*N*-oxide) has exhibited many emergent functions such as mitochondria targeting,^33^ cell-penetrating,^34^ drug delivery,^35,36^ etc. To date, the synthesis of poly­(*N*-oxide) mostly relies on free radical polymerization to afford hydrogels^30,37,38^ or postpolymerization modification of tertiary amine-containing polymer precursors,^33–35,39^ resulting in poor control over the extent of oxidation and polymer molecular weight. To the best of our knowledge, the direct synthesis of poly­(*N*-oxide) from the corresponding *N*-oxide monomer with RDRP techniques is rather limited.^40,41^ Our group reported *N*-oxide-(*N,N*-diethylamino)­ethyl methacrylate (ODEMA) through thermally initiated RAFT polymerization. The obtained polymers have demonstrated excellent biofouling resistance against proteins, bacteria, and human cells.^42^ However, the methacrylate backbone is known to lose its antifouling performance upon standard thermal or steam sterilization due to hydrolysis of the ester bond, leading to the reactivation of bioadhesion and biofilm formation. Chang and coworkers reported that zwitterionic polymer thermal stability can be significantly enhanced when the methacrylamide backbone is used instead of the methacrylate backbone, thus allowing the use of a high-temperature sterilization process (e.g., autoclave), and the sterilized materials maintained good antifouling properties due to the better hydrolytic stability of amide in comparison to that of ester. In addition, the (meth)­acrylamide backbone is more hydrophilic and exhibits stronger inter- and intramolecular hydrogen bonding, which is anticipated to facilitate the formation of a hydration layer that is crucial to fouling resistance.^43^

Noting that current reports on *N*-oxide polymers are mostly acrylate- or methacrylate-based *N*-oxide,^40–42,44^ (meth)­acrylamide-based poly­(*N*-oxide) is rather limited. In our hands, we found that direct polymerization of (meth)­acrylamide-based *N*-oxide monomers could not be achieved with thermally initiated RAFT polymerization due to unexpected side reactions in the presence of both dithioester and trithiocarbonate chain transfer agents at elevated temperatures. To this end, we exploited the utilities of ZnTPP and ZnTPS_4_ as photocatalysts for PET-RAFT of (meth)­acrylamide-based *N*-oxide monomers, which underwent controlled polymerization of poly­(*N*-oxide) under red-light irradiation. The balance of catalyst solubility and water content imparts control over polymerizations, and the polymer dispersity can be varied by the amount of DMSO and water. The livingness of ZnTPP- and ZnTPS_4_-mediated PET-RAFT was examined by chain-extension experiments. Furthermore, oxygen tolerance, monomer scope, and the feasibility to obtain ultrahigh molecular weight (UHMW) polymers were thoroughly investigated. This work provides a robust and accessible platform for preparing poly­(*N*-oxide) and other hydrophilic polymers with controlled molecular weight, enabling further advanced studies in surface coatings, bioconjugations, and biomaterials designs.

## 2 Results and Discussion

A series of *N*-oxide monomers featuring (meth)­acrylamide as the polymerizable groups were prepared by oxidizing the corresponding tertiary amine-containing monomers with *meta*-chloroperoxybenzoic acid (*m*CPBA) to afford *N*-oxide-3-(*N*,*N*-dimethylamino)­propyl methacrylamide (ODMMAm), *N*-oxide-2-(*N,N*-diethylamino)­ethyl methacrylamide (ODEMAm), and *N*-oxide-3-(*N,N*-dimethylamino)­propylacrylamide (ODMAm) by adapting the same synthetic approach as the previously reported *N*-oxide-2-(*N,N*-diethylamino)­ethyl methacrylate (ODEMA).^42,44^ Such an approach avoids the use of oxygen gas as the reaction atmosphere. The monomers were then purified by column chromatography using neutral aluminum oxide (Al_2_O_3_) as the stationary phase. The structural identities of these *N*-oxide monomers were confirmed by ^1^H and ^13^C NMR spectroscopies and high-resolution mass spectrometry (HRMS) (Section 4, Supporting Information), and the chemical structures of CTAs and other hydrophilic monomers used in this study are shown in Figure 1.

**Figure 1:**
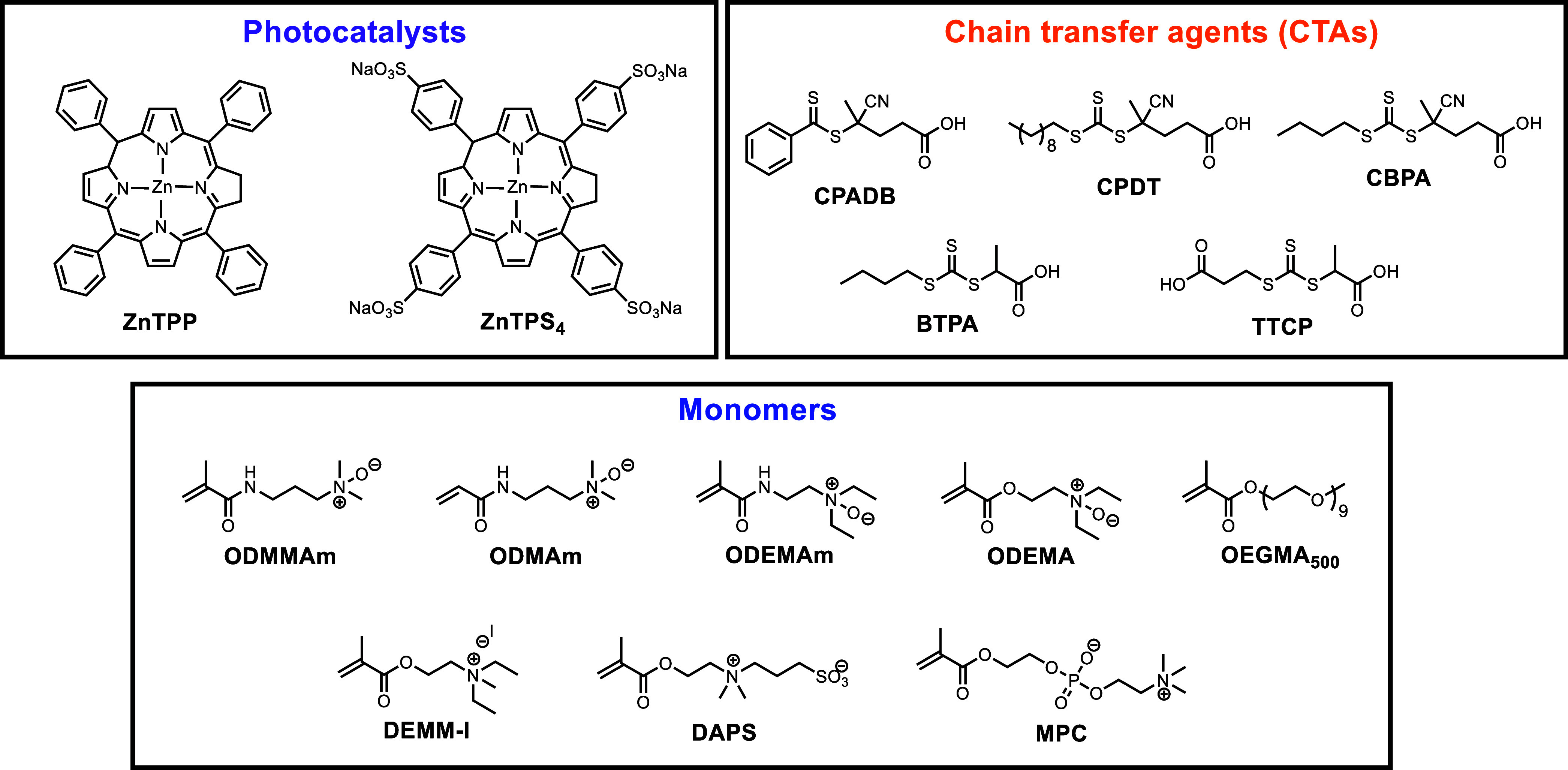
Photocatalysts (PCs), chain transfer agents (CTAs), and monomers were investigated in this work.

Initial attempts to conduct thermally initiated RAFT polymerization of *N*-oxide-3-(*N*,*N*-dimethylamino)­propyl methacrylamide (ODMMAm) with 4,4’-azobis­(4-cyanovaleric acid) (ACVA) as the radical initiator were problematic, despite the same conditions being found suitable for *N*-oxide containing methacrylate ODEMA, as described in our previous publication.^42^ The polymerization of ODMMAm was conducted with a ratio of [M]:[CPADB]:[I] = 200:1:0.1 and a monomer concentration of 1.5 M in DMSO at 70 °C for 12 h. GPC analysis showed a monomodal distribution along with a number-average molecular weight (*M*_n_) of 28.2 kDa and a molecular weight distribution (Đ) of 1.80. The color of the polymerization mixture turned yellow over time, indicating a loss of CTA functionality (Figure S3). Given that there was a low monomer conversion of 13%, as determined by ^1^H NMR, along with the observation of a large discrepancy of 453% between the targeted and GPC-derived molecular weights, the inability to conduct RAFT polymerization of *N*-oxide-containing methacrylamide was unexpected (Table S2, entry 1). Although the GPC-derived molecular weight often deviates from the theoretical molecular weight because the correlation factor between hydrodynamic volume and molecular weight varies for different polymers, such a large discrepancy in molecular weight is likely to be abnormal and a Đ of 1.80 further suggests the loss of control, which is not as expected for a typical RAFT polymerization.

The thermal stability of ODMMAm in DMSO-*d*_6_ was then interrogated with variable temperature ^1^H NMR experiments. We did not observe any significant changes in proton signals at the polymerization temperature, and the ODMMAm is stable up to 90 °C (Figure S4). We observed a downfield shift of protons in the aromatic region corresponding to the RAFT agent when heating a DMSO-*d*_6_ solution of ODMMAm in the presence of 0.2 equiv of CPADB at 70 °C for 2h (Figure S5). An additional experiment was conducted by heating ODMMAm and CPADB in methanol at 70 °C for 12 h. The crude reaction mixture was directly analyzed by high-resolution liquid chromatography-tandem mass spectrometry (HPLC-MS). The HPLC chromatogram (Figure S6) shows a large fraction of ODMMAm (46.6%area, peak 1) remained after heating, an adduct of deoxygenated ODMMAm and CPADB (15.3%area, peak 2), and the Cope elimination products of ODMMAm (12.1%area, peak 6) were observed, which are consistent with the previous reports on Cope elimination of *N*-oxide that afford alkene and dialkylhydroxylamine byproducts at elevated temperature.^45–47^ The MS spectra of peaks 3, 4, and 5 were difficult to speculate on because the *m*/*z* values of these fragments failed to match the predicted chemical structures, and the formation of these new species could be attributed to the electrospray ionization (ESI) step.

The poor control of methacrylamide-based monomers by RAFT polymerization has been attributed to the degradation of the RAFT agent due to the intramolecular nucleophilic attack of the amide proton at high temperature.^48,49^ Therefore, polymerization was conducted by using 2,2’-azobis­[2-(2-imidazolin-2-yl)­propane]­dihydrochloride (VA-044) as the radical initiator, allowing the polymerization to be conducted at 45 °C. However, the polymerization did not proceed at this temperature (Table S2, entry 2). According to other literature precedents, the control over molecular weight and molecular weight distribution of hydrophilic monomers (i.e., 2-hydroxypropyl methacrylamide) could be improved by using water as the solvent for RAFT polymerization due to the disruption of intra- and intermolecular interactions.^50,51^ Additional experiments were performed using ACVA or VA-044 in water with the same feeding ratio as that mentioned above. The polymerization using ACVA afforded a polymer with a monomer conversion of 80%, an *M*_n_ of 31.9 kDa, a Đ of 3.17, and a deviation of 6% from the theoretical molecular weight (Table S2, entry 3). The GPC trace shows a broad bimodal distribution that could be attributed to undesired side reactions such as chain transfer, couplings, and terminations during polymerization at high temperature (Figure S7). Meanwhile, the polymerization using VA-044 afforded a polymer with a monomer conversion of 70%, an *M*_n_ of 28.9 kDa, a Đ of 1.54, and a deviation of 10% from the theoretical molecular weight (Table S2, entry 4). These observations suggest that conventional RAFT polymerization is not compatible with methacrylamide-based *N*-oxide monomers. In addition, the acrylamide-based *N*-oxide monomer also failed to produce well-defined polymers regardless of the choice of CTAs.

We then utilized the room-temperature PET-RAFT protocol developed by Boyer and coworkers, which has emerged as a powerful platform to achieve controlled polymerization for a wide range of monomers. ZnTPP was adopted as the PC due to its excellent photocatalytic activities and commercial availability in the polymerization of (meth)­acrylates and (meth)­acrylamides under visible-light irradiation.^21^ The initial photopolymerization was conducted at the ratio of [M]:[CPADB]:[PC] = 200:1:0.01 by using ODMMAm as a monomer and 4-cyano-4-(thiobenzoylthio)­pentanoic acid (CPADB) as a RAFT agent. The initial monomer concentration was 3 M in DMSO, and a catalyst loading of [PC]:[M] = 50 ppm, where the final photocatalyst concentration is 50 ppm relative to the monomer. The scintillation vial was degassed by bubbling with N_2_ for 15 min. The vial was then placed 10 cm away from the light source and irradiated with red LED light (λ_max_= 625 nm, *I* = 35 mW/cm^2^) for 4 h. The reaction set up is shown in Figure S8. The polymerizations were also performed in mixtures of water and DMSO, and the polymerization results are summarized in Table 1.

**Table 1:** PET-RAFT Polymerization of ODMMAm under Red-Light Irradiation for 4 h^a^

Entry	PC	Solvent (% vol/vol)	α (%)^b^	*M*_n,th_ ^c^ (kDa)	*M*_n,GPC_ ^d^ (kDa)	Đ^d^	Dev.^e^
1	ZnTPP	DMSO	20	7.7	35.8	2.06	365%
2		10% H_2_O/DMSO	43	16.3	43.7	2.03	168%
3		30% H_2_O/DMSO	66	24.9	37.5	1.51	51%
4	ZnTPS_4_	30% H_2_O/DMSO	59	22.3	51.8	2.46	132%
5		50% H_2_O/DMSO	50	19.0	27.0	1.13	42%
6		70% H_2_O/DMSO	54	20.5	29.0	1.11	41%
7		H_2_O	85	31.8	35.1	1.15	10%

a Polymerization was carried out under deoxygenated conditions at the initial ratio of [M]:[CPADB]:[PC] = 200:1:0.01, with a catalyst loading of [PC]:[M] = 50 ppm and a monomer concentration [M] = 3M.

b Determined by ^1^H NMR spectroscopy.

c Theoretical molecular weight was calculated using the following equation: *M*_n,th_= [*M*_o_]/[CTA] × α × *M*_w,monomer_ + *M*_w,CTA_, where [*M*_o_], [CTA], α, *M*_w,monomer_, and *M*_w,CTA_ correspond to the initial monomer concentration, initial CTA concentration, monomer conversion determined by ^1^H NMR, molar mass of the monomer, and molar mass of the CTA.

d Molecular weight (*M*_n,GPC_) and dispersity (Đ) were determined by GPC analysis (TFE as eluent) calibrated using PMMA standards

e Deviation (Dev.)= |*M*_n,th_ – *M*_n,GPC_|/*M*_n,th_ × 100%.

The polymerization performed in DMSO gave a monomer conversion of 20%, as determined by ^1^H NMR after 4 h of irradiation, affording a polymer with an *M*_n_ of 35.8 kDa, a Đ of 2.06, and a deviation of 365% from the theoretical molecular weight (Table 1, entry 1). In the presence of 10 vol % H_2_O in DMSO, a monomer conversion of 43% was achieved, affording a polymer with an *M*_n_ of 43.7 kDa, a Đ of 2.03 (entry 2), and a deviation of 168%. Further increasing the water content to 30 vol % H_2_O in DMSO boosts the monomer conversion to 66%, affording a polymer with an *M*_n_ of 37.5 kDa, a Đ of 1.51, and a deviation of 51% (entry 3). Two additional photopolymerization experiments were performed in the absence of either the RAFT agent or ZnTPP, and zero conversion rules out the possibility of a photoiniferter polymerization mechanism and radical generation caused by the photocatalyst under red-light irradiation (Table S3, entries 1–2). It is worth noting that the ZnTPP photocatalyst is poorly soluble in 30% H_2_O/DMSO (v/v), appearing as a slightly cloudy solution during polymerization (Figure S9) and the solution color is different than that of the reaction mixture with 10–20% H_2_O/DMSO (v/v). Kinetic studies of polymerizations done in DMSO and 10 vol % H_2_O/DMSO revealed that early termination occurred after 30 min of polymerization, leading to the low conversion and large deviation from the theoretical molecular weight under such solvent systems (Figure S10a). In contrast, pseudo-first-order kinetics with an apparent rate of 0.361 h^–1^ was achieved by using 30 vol % H_2_O/DMSO. Controlled polymerization was achieved in the first hour of polymerization, as indicated by Đ < 1.2; however, a gradual increase in Đ was observed after 2 h of polymerization, and the Đ reached 1.9 at the end of polymerization (Figure S10b).

To overcome this issue, a water-soluble zinc porphyrin derivative, zinc­(II) *meso*-tetra­(4-sulfonatophenyl) porphyrin tetrasodium (ZnTPS_4_), was evaluated as a PC for PET-RAFT.^53^ The photopolymerization experiments with ZnTPS_4_ were performed under the above-mentioned conditions. First, the polymerization in 30 vol % H_2_O/DMSO gave a monomer conversion of 59%, affording a polymer with an *M*_n_ of 51.8 kDa and a Đ of 2.46, which deviates 132% from *M*_n,th_, suggesting that the aqueous-soluble ZnTPS_4_ disfavors the low aqueous environment used (Table 1, entry 4). Upon increasing the aqueous content to over 50 vol % H_2_O/DMSO, a critical change in Đ was observed, indicating the important role of H_2_O to achieve controlled radical polymerization of *N*-oxide monomer ODMMAm. Poly­(ODMMAm) with an *M*_n_ of 27.0 kDa and a Đ of 1.13 was obtained at a monomer conversion of 50% after 4 h when the polymerization was performed in 50 vol % H_2_O/DMSO (Table 1, entry 5). Further increase in the volume ratio of water to 70 vol % results in a monomer conversion of 54% within the same period, affording a polymer with an *M*_n_ of 29.0 kDa and a Đ of 1.11 (Table 1, entry 6). A control experiment showed no monomer conversion in the absence of ZnTPS_4_, confirming that the polymerization happened through the PET-RAFT mechanism (Table S3, entry 3). In addition, the control experiment conducted in the absence of the RAFT agent showed negligible monomer conversion, which rules out the possibility of radical generation by the PC under red-light irradiation (Table S3, entry 4). When polymerization was conducted in a fully aqueous environment, the monomer conversion increased to 85%, and an improvement in control over the molecular weight was achieved (*M*_n,th_= 31.8 kDa, *M*_n,GPC_= 35.1 kDa; Table 1, entry 7). These results suggest that optimization of solvent composition and catalyst solubility is important for achieving controlled PET-RAFT polymerization of ODMMAm. Because CPADB is not fully dissolved in water at the beginning of polymerization, we chose 70% H_2_O/DMSO as the solvent system for later studies to ensure the solubility of the RAFT agent.

The efficiency of photochemical reactions is dependent on the light absorption and light-to-chemical energy conversion capabilities of PCs. The broad absorption spectrum of ZnTPS_4_ (Figure S11) prompts us to investigate the photopolymerization efficacy under irradiation by green (λ_max_= 520 nm) and blue (λ_max_= 450 nm) LEDs. The results of photopolymerization are summarized in Table 2. In all cases, high monomer conversions (50–90%) were observed, along with excellent control over the molecular weight (1.10 ≤ Đ ≤ 1.30) (Table 2, entries 1–3).

**Table 2:** Polymerization of ODMMAm Using Different Light wavelengths^a^

Entry	[PC]/[M] (ppm)	Light	*I* (mW/cm^2^)	λ_max_ (nm)	α (%)^b^	*M*_n,th_ ^c^ (kDa)	*M*_n,GPC_ ^d^ (kDa)	Đ^d^	Dev.^e^
1^f^	50	Red	35	625	54	20.5	29.0	1.11	41%
2		Green	28	520	61	23.0	33.8	1.15	47%
3		Blue	23	450	87	32.7	40.9	1.28	25%

a Reaction conditions: [ODMMAm]:[CPADB]:[ZnTPS_4_] = 200:1:*x*, [ODMMAm] = 3 M in 70% H_2_O/DMSO, irradiated for 4 h under different LEDs.

b Determined by ^1^H NMR spectroscopy.

c Theoretical molecular weight was calculated using the following equation: *M*_n,th_= [*M*_o_]/[CTA] × α × *M*_w,monomer_ + *M*_w,CTA_, where [*M*_o_], [CTA], α, *M*_w,monomer_, and *M*_w,CTA_ correspond to the initial monomer concentration, initial CTA concentration, monomer conversion determined by ^1^H NMR, molar mass of the monomer, and molar mass of the CTA.

d Molecular weight (*M*_n,GPC_) and dispersity (Đ) were determined by GPC analysis (TFE as eluent) calibrated using PMMA standards.

e Deviation (Dev.)= |*M*_n,th_ – *M*_n,GPC_|/*M*_n,th_ × 100%.

f Taken from Table 1, entry 6.

Since there are many previous reports on the photoiniferter RAFT polymerization (PI-RAFT) by the direct activation of CTA under blue- and green-light irradiation,^54–56^ additional photopolymerizations of ODMMAm were performed in the absence of ZnTPS_4_ to eliminate the participation of dual mechanisms, i.e., PI-RAFT and PET-RAFT, at shorter irradiation wavelengths. A monomer conversion of 30% was observed after 4 h of irradiation under green light, yielding a polymer with an *M*_n_ of 19.7 kDa and a Đ of 1.12 (Table S4, entry 2). In addition, a higher monomer conversion of 53% was achieved under blue-light irradiation, affording a polymer with an*M*_n_ of 30.7 kDa and a Đ of 1.22 (Table S4, entry 3). As mentioned in the previous section, red-light irradiation could not induce the polymerization of ODMMAm in the absence of ZnTPS_4_ (Table S4, entry 1). These results are consistent with previous findings on the PI-RAFT; we continued to employ red light (λ_max_= 625 nm) in the later studies to prevent potential interference from the PI-RAFT mechanism.

As PET-RAFT polymerization can be carried out under two different quenching pathways, namely an oxidative quenching pathway (OQP) and a reductive quenching pathway (RQP), as shown in Figure 2.^10,57,58^ In which, the OQP involves electron transfer from an excited-state PC to a ground-state CTA, leading to the formation of a radical cation PC (PC^•+^) and an anionic intermediate of CTA, which subsequently undergoes fragmentation to generate a propagating radical (Figure 2A).^59,60^ Meanwhile, the RQP involves the reduction of an excited-state PC in the presence of a sacrificial reducing agent (tertiary amine, ascorbic acid, etc.). A radical anion of PC (PC^•–^), formed through an electron transfer process from the reducing agent to the excited-state PC, undergoes a single electron transfer (SET) to the CTA to generate propagating radical species (Figure 2B).^61–63^ We first conducted a kinetics study under OQP conditions to gain insight into the efficiency of the PET-RAFT process. As shown in Figure 3a, polymerization conducted under red-light irradiation in 50% H_2_O/DMSO and 70% H_2_O/DMSO has apparent rates of 0.219 h^–1^ and 0.313 h^–1^, respectively. A linear growth in molecular weight with respect to time was observed in both cases, and the Đ values of the kinetic samples remained below 1.2, indicating excellent control of the polymerization process (Figure 3b and Figure S12). Alternatively, we performed an additional kinetics study to evaluate the efficiency of RQP in the presence of a tertiary amine (i.e., triethylamine or triethanolamine (TEOA)). First, 10 equiv of triethylamine relative to CTA were used as a scarifying agent, which gave a monomer conversion of 98%, affording a polymer with an *M*_n_ of 55.5 kDa, a Đ of 1.44, and a deviation of 52% (Table S5, entry 1). Meanwhile, the use of TEOA afforded a polymer with a monomer conversion of 81%, an *M*_n_ of 39.9 kDa, a Đ of 1.16, and a deviation of 32% (Table S5, entry 2). This can be attributed to the difference in radical stability of the amine radical anions generated under the RQP catalytic cycle.^64^ A control experiment performed at the ratio of [ODMMAm]:[CPADB]:[TEOA] = 200:1:5 and in the absence of ZnTPS_4_ photocatalyst showed no monomer conversion, suggesting that TEOA cannot be activated by red-light irradiation (entry 1, Table S6). As recent reports on PET-RAFT polymerization under visible light indicate the generation of α-aminoalkyl radicals (TEOA^•^) or aldehyde-bearing α-aminoalkyl radical (A-TEOA^•^) species,^65,66^ a series of control experiments in the absence of the RAFT agent were conducted under deoxygenation and open-to-air conditions. No polymer was obtained, as revealed by ^1^H NMR spectra and GPC analysis after 4 h of irradiation (entries 2 and 3, Table S6). In addition, Figure S13 shows the ^1^H NMR spectra (DMSO-*d*_6_) of the crude polymerization mixtures obtained with and without deoxygenation. The absence of a characteristic aldehyde peak (8–10 ppm) suggests that A-TEOA^•^ is unlikely to be present in our reaction system. Taking all results together, it indicates that methacrylamide polymer chains cannot be initiated by either TEOA^•^ or A-TEOA^•^ under red-light irradiation. In addition, the effect of TEOA equivalence was also investigated, and the results show that there was no significant difference in monomer conversion at 5, 10, and 15 equiv of TEOA in the polymerization mixtures (Table S5, entries 3–4). Therefore, the optimal polymerization condition for ODMMAm was determined as [M]:[CPADB]:[PC]:[TEOA] = 200:1:0.01:5 under red-light irradiation.

**Figure 2:**
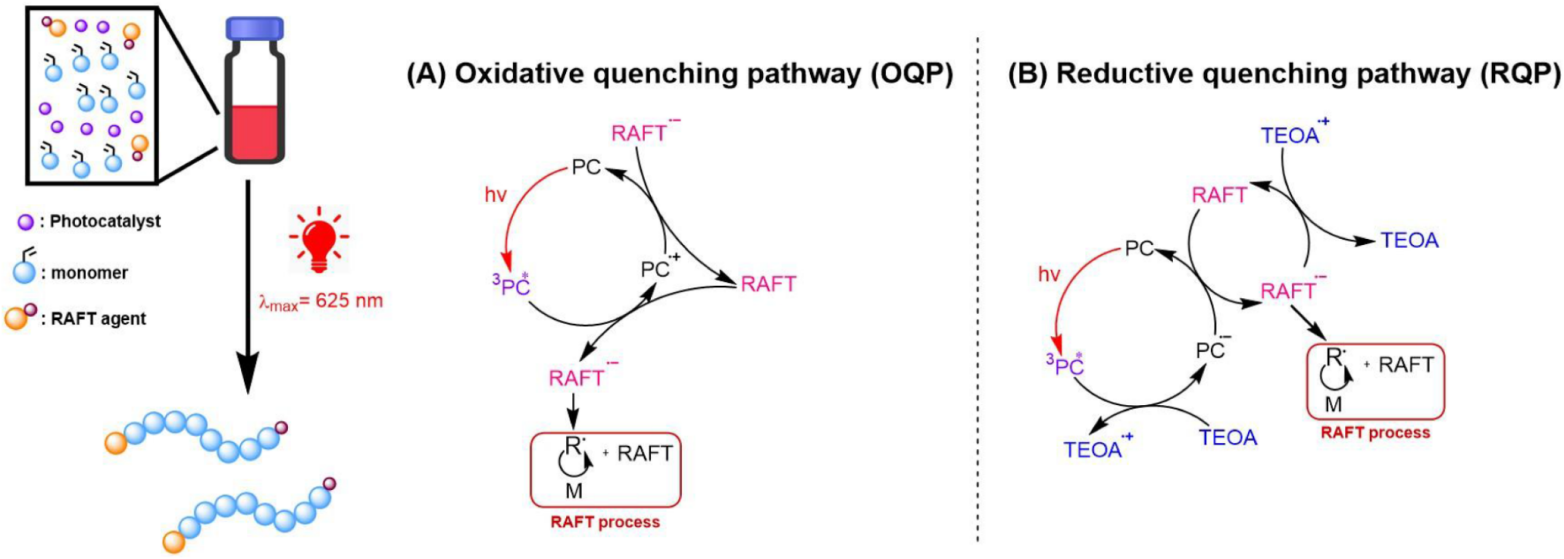
A schematic illustrates the red-light-driven PET-RAFT polymerization of *N*-oxide monomer via an oxidative quenching pathway (OQP, a) and a reductive quenching pathway (RQP, b).

**Figure 3:**
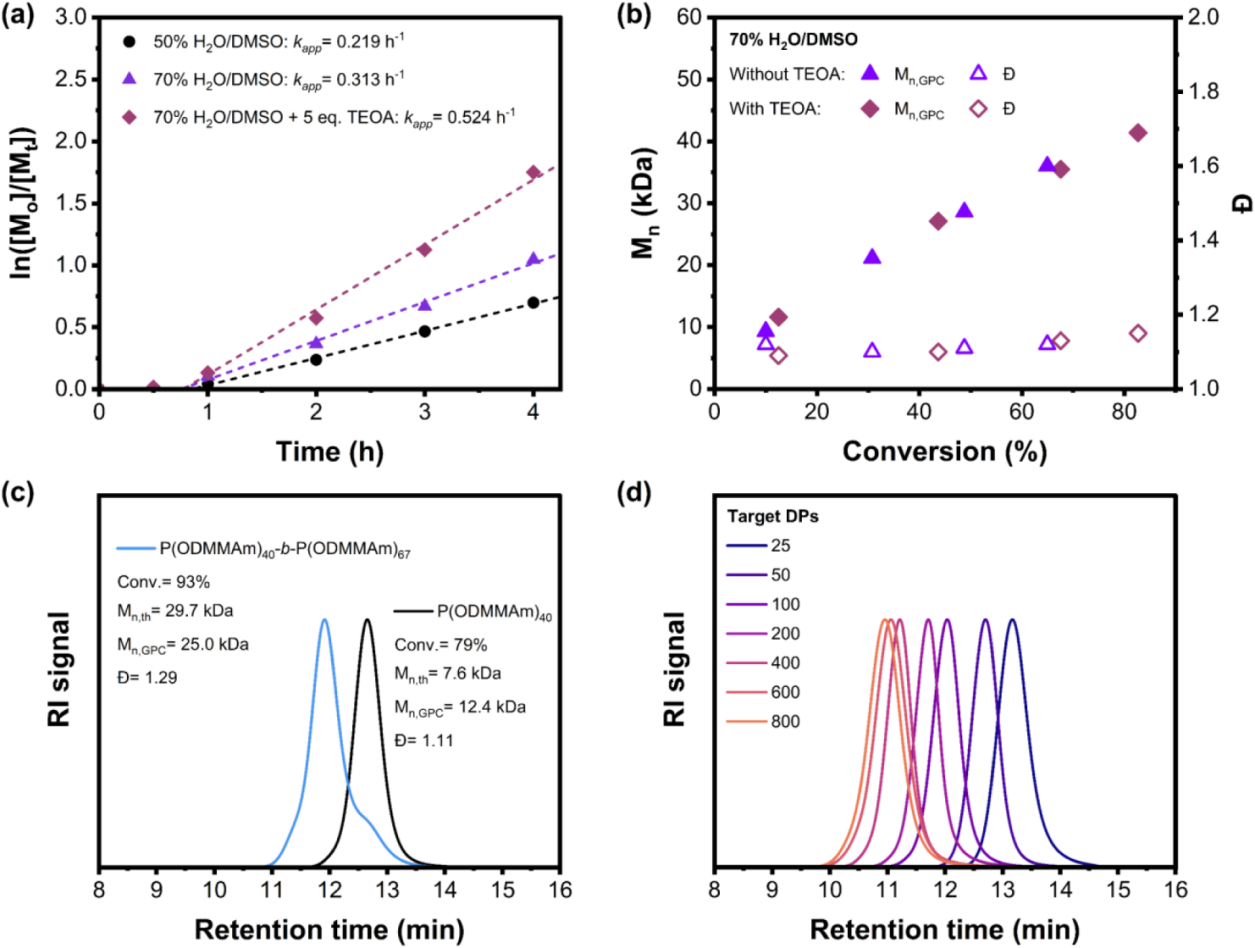
(a) Kinetics study of PET-RAFT polymerization of ODMMAm in the absence of oxygen at room temperature with CPADB as the chain transfer agent, ZnTPS_4_ as the photocatalyst under red- light irradiation, using the ratio of [M]:[CPADB]:[PC] = 200:1:0.01 and [PC]:[M] = 50 ppm in different solvent systems. (b) Evolution of *M*_n_ and Đ versus monomer conversion in the absence and presence of TEOA as a scarifying electron donor;. (c) GPC traces of P­(ODMMAm) macro-CTAs and their diblock copolymer synthesized via *in situ* chain extension in 70% H_2_O/DMSO;. (d) GPC traces of P­(ODMMAm) representing different degrees of polymerization synthesized via ZnTPS_4_-mediated PET-RAFT polymerization.

To elucidate the livingness of the synthesized polymer, we carried out the PET-RAFT polymerization of ODMMAm with a feed ratio of [M]:[CPADB]:[ZnTPS_4_]:[TEOA] = 50:1:0.0025:5 and an initial monomer concentration of 3 M. After 4 h of irradiation under red light, a monomer conversion of 79% was achieved, as determined by ^1^H NMR spectroscopy, affording a polymer with an *M*_n_ of 12.4 kDa, and a Đ of 1.11, namely P­(ODMMAm)_40_ and this macro-CTA was later used in block copolymerization. A chain-extension experiment was carried out by adding additional ODMMAm with the target DP of 50 to the crude macro-CTA solution, and the feed ratio was [M]:[macro-CTA] = 67:1, as determined by ^1^H NMR spectroscopy (Figure S14). The resulting block copolymer was named as P­(ODMMAm)_40_-*b*-P­(ODMMAm)_67_. After another 4 h of irradiation, ^1^H NMR spectroscopy revealed a monomer conversion of 93%. The GPC traces show a significant shift toward shorter retention time, confirming that the ZnTPS_4_-mediated PET-RAFT polymerization can produce a polymer with sufficient chain-end functionality (Figure 3c). The resultant block copolymer P­(ODMMAm)_40_-*b*-P­(ODMMAm)_67_ has an *M*_n_ of 25.0 kDa and a Đ of 1.29. The increase in molecular weight distribution and the appearance of a small lower molecular weight shoulder peak can be attributed to the residual polymer chains without CTA end groups from the previous polymerization process, as well as the formation of dead chains during the chain-extension process. The blocking efficiency was estimated to be 44.2% by deconvolution of the GPC peak (Figure S15 and Equation S1), where the peak at a higher molecular weight, possibly formed by chain coupling, was not taken into calculation. The blocking efficiency can be improved by further optimization of polymerization conditions, such as RAFT agent, reaction time, solvent, etc. Additional block copolymerization was performed by using 4-cyano-4-(((decylthio)­carbonothioyl)­thio)­pentanoic acid (CPDT) as a RAFT agent. The macro-CTA was synthesized with a feed ratio of [ODMMAm]:[CPDT]:[ZnTPS_4_]:[TEOA] = 50:1:0.0025:5 at a monomer concentration of 3.0 M in 70% H_2_O/DMSO and irradiated for 2 h under red light. The resulting macro-CTA, namely P­(ODMMAm)_50_, gave a monomer conversion of 78%, an *M*_n_ of 19.2 kDa, and a Đ of 1.22. Subsequently, fresh monomer was added to unpurified P­(ODMMAm)_50_ at the target DP of 50. The whole mixture continued irradiating with red light for 2 h to afford a block copolymer, namely P­(ODMMAm)_50_-*b*-P­(ODMMAm)_50_, with an *M*_n_ of 32.8 kDa and a Đ of 1.23 at 94% monomer conversion. A clean shift toward the higher molecular weight region without significant tailing confirms the good end-group fidelity of the trithiocarbonate RAFT agent on our polymer chains (Figure S16). These results highlight the ability of the ZnTPS_4_-mediated PET-RAFT polymerization system in the synthesis of block copolymer under ambient conditions.

The ability to achieve well-defined polymers over a wide range of molecular weights is one of the important properties of the RDRP technique. To evaluate the scope of molecular weight control of poly­(*N*-oxide), which has not been reported in previous literature, polymerization with various targeted degrees of polymerization from 25 to 800 was performed by varying the ratio of monomer and CTA at 50 ppm of ZnTPS_4_ (Figure S17). Remarkably, Table S7 shows that the controlled polymerization was able to achieve a DP target range from 25 to 800 without a significant difference in monomer conversion, suggesting excellent reproducibility of the ZnTPS_4_ system in PET-RAFT polymerization of methacrylamide-based *N*-oxide. A narrow molecular weight distribution (Đ < 1.3) was maintained with the target DP ≤ 800 without significant tailing, as observed in these GPC traces, which are shown in Figure 3d. Considering the growing interest in oxygen-tolerant polymerization, we also tested our polymerization in the absence of any external deoxygenation methods by simply minimizing the headspace volume to lower the level of initial oxygen. The polymerization was conducted with a feed ratio of [ODMMAm]:[CPADB]:[ZnTPS_4_]:[TEOA] = 200:1:0.01:5 in a capped vial without deoxygenation and in an open vial (Figure S18). Pleasingly, a well-controlled polymerization took place in both cases, resulting in a polymer with an *M*_n_ of 29.8 kDa, a Đ of 1.15 at 77% monomer conversion, and an *M*_n_ of 31.5 kDa, a Đ of 1.14 at 77% monomer conversion for the polymerization conducted under open-to-air and capped vial (no deoxygenation), respectively (Table S8 and Figure S19). Successful polymerization under open-to-air conditions can be attributed to the capture of singlet oxygen (^1^O_2_) by DMSO, which is generated from the energy transfer process between ground-state oxygen (^3^O_2_) and the photocatalyst through triplet–triplet annihilation (TTA).^67^ These results demonstrate the oxygen tolerance of the ZnTPS_4_-mediated PET-RAFT polymerization system under red-light irradiation, thus simplifying the polymerization procedure for future applications, i.e., surface-initiated polymerization, synthesis of protein–polymer hybrid materials, etc.

Recently, the Hoogenboom group revealed that the backbone chemistry of ZP plays a crucial role in polymer-cellular interactions. Typically, methacrylamide-derived polymers have a high association with both breast cancer cells and noncancerous dendritic cells. In contrast, acrylamide-derived polymers are highly selective for only breast cancer cells.^68^ These findings suggest that having a universal polymerization technique to achieve controlled polymerization for both methacrylamide- and acrylamide-based *N*-oxide monomers is very important due to the increasing use of poly­(*N*-oxide) as a platform for drug delivery in recent years.^34,40,41,44,69^ Therefore, we further evaluated the compatibility of ZnTPS_4_-mediated PET-RAFT polymerization with the acrylamide-based *N*-oxide monomer ODMAm. Initially, we performed photopolymerization with a feed ratio of [ODMAm]:[CTA]:[PC]:[TEOA] = 200:1:0.01:5 and a monomer concentration of 3 M in 70% H_2_O/DMSO by using *N*-oxide-3-(*N,N*-dimethylamino)­propylacrylamide (ODMAm) as the model monomer, CPDT as the chain transfer agent, and ZnTPS_4_ as the photocatalyst. However, we observed the formation of stable foam during the degassing process, either by bubbling N_2_ gas or freeze–pump–thaw (Figure S20). This can be explained by the amphiphilicity of CPDT, the use of aqueous solvent mixtures, and the *N*-oxide compounds have been reported as surfactants for many pharmaceutical products and detergents.^70,71^ To overcome the incompatibility issue, we replaced the hydrophobic CPDT with three hydrophilic trithiocarbonate CTAs, i.e., 4-cyano-4-(((butylthio)­carbonothioyl)­thio)­pentanoic acid (CBPA), 2-(((butylsulfanyl)­carbothioyl)­sulfanyl)­propanoic acid (BTPA), and 3-((((1-carboxyethyl)­thio)­carbonothioyl)­thio)­propanoic acid (TTCP). Table 3 summarizes the results of PET-RAFT polymerization using these CTAs. Even though all polymerizations reached nearly quantitative monomer conversion in an hour, a good control over polymerization was achieved, except for BTPA, which gave a slightly larger Đ of 1.36 (Table 3, entry 1). In contrast, excellent control over polymerization was achieved when using CBPA as a chain transfer agent, affording a polymer with an *M*_n_ of 38.4 kDa, a Đ of 1.19, and a deviation of 14% (Table 3, entry 3 and Figure S21). Control experiments performed in the absence of either the photocatalyst or the CTA gave negligible monomer conversion, indicating that the photoiniferter mechanism and direct activation of the monomer are not feasible (entries 4 and 5).

**Table 3:** PET-RAFT Polymerization of ODMAm under Red-Light irradiation^a^

Entry	[M]:[CTA]:[ZnTPS_4_]	CTA	α (%)^b^	*M*_n,th_ ^c^ (kDa)	*M*_n,GPC_ ^d^ (kDa)	Đ^d^	Dev.^e^
1	200:1:0.01	BTPA	99	34.4	38.7	1.36	13%
2	200:1:0.01	TTCP	98	34.3	40.1	1.27	17%
3	200:1:0.01	CBPA	96	33.5	38.4	1.19	14%
4	200:1:0	CBPA	0	---	---	---	---
5	200:0:0.01	CBPA	2	---	---	---	---

a Polymerization was carried out under deoxygenated conditions with a catalyst loading of [ZnTPS_4_]:[M] = 50 ppm and a monomer concentration [M] = 3 M. The polymerization mixture was irradiated with red light for 1 h.

b Determined by ^1^H NMR spectroscopy.

c Theoretical molecular weight was calculated using the following equation: *M*_n,th_= [*M*_o_]/[CTA] × α × *M*_w,monomer_ + *M*_w,CTA_, where [*M*_o_], [CTA], α, *M*_w,monomer_, and *M*_w,CTA_ correspond to the initial monomer concentration, initial CTA concentration, monomer conversion determined by ^1^H NMR, molar mass of the monomer, and molar mass of the CTA.

d Molecular weight (*M*_n,GPC_) and dispersity (Đ) were determined by GPC analysis (TFE as eluent) calibrated using PMMA standards.

e Deviation (Dev.)= |*M*_n,th_ – *M*_n,GPC_|/*M*_n,th_ × 100%.

Figure 4a shows the kinetics study results of ZnTPS_4_-mediated PET-RAFT polymerization of ODMAm with a DP_target_ of 200 in the presence and absence of TEOA. Both polymerizations showed an induction period of 30–40 min, depending on the presence or absence of TEOA in the reaction system. Pseudo-first-order kinetics were observed, with the apparent rate of 7.244 h^–1^ in the absence of TEOA and 9.333 h^–1^ in the presence of TEOA. Remarkably, the apparent rate of ODMAm was 18 times higher than that of ODMMAm under the same reaction conditions. A linear growth of molecular weight versus monomer conversion was observed in both cases, while the Đ decreased as a function of monomer conversion and reached a Đ of less than 1.2 at the end of polymerization, which can be attributed to the slow equilibrium between dormant species and RAFT intermediates (Figure 4b and Figure S22). Even though the trithiocarbonate RAFT agent is known to have better reactivity and selectivity in porphyrin-catalyzed PET-RAFT polymerization systems due to the specific coordination of trithiocarbonate with zinc porphyrins,^21^ such a huge difference in apparent rate has not been reported before. Therefore, a series of control experiments were performed to reveal the mechanistic insight into the rate acceleration effect in PET-RAFT polymerization of ODMAm. First, the polymerization performed at the feed ratio of [ODMAm]:[CBPA]:[TEOA] = 200:1:5 and in the absence of ZnTPS_4_ gave no monomer conversion after 1 h of irradiation, as determined by both ^1^H NMR spectroscopy and GPC analysis (entry 4, Table S6). Other control experiments were performed in the absence of the RAFT agent while keeping the ratio [ODMAm]:[ZnTPS_4_]:[TEOA] = 200:0.01:5 under deoxygenation and open-to-air conditions (entries 5–6, Table S6). There are no characteristic signals of the aldehyde proton that can be observed in the ^1^H NMR spectrum of the crude polymerization mixture, suggesting that the A-TEOA^•^ is not present in our polymerization system. However, a trace amount of ultrahigh molecular weight polymer was obtained, even though the ^1^H NMR spectrum showed no or negligible monomer conversion (Figure S23); the reaction mixture appeared highly viscous. The polymers produced under deoxygenation and open-to-air conditions are reported with a peak molecular weight (*M*_p_) of 626.3 and 667.7 kDa, since a large area of the peak is out of our column exclusion limit (Figure S24). The zero monomer conversion in ^1^H NMR can be attributed to the ultralow concentration of the TEOA^•^ in the system, where only a few polymer chains are successfully initiated. The initiating radical (TEOA^•^) was generated through an energy/electron transfer process between TEOA and ZnTPS_4_ under red-light irradiation. Such radical generation will stop when all ZnTPS_4_ are converted into ZnTPS_4_^•–^, which cannot be reduced back to the ground-state ZnTPS_4_ in the absence of the RAFT agent (Figure 2B). Since the loading of ZnTPS_4_ in the control experiments was 0.005 mol % relative to the monomer, which correlates to a target DP of 20,000 and a theoretical molecular weight of 3360 kDa. Taken together, the high apparent rate of ODMAm over ODMMAm can be attributed to two reasons: (i) highly efficient activation of the trithiocarbonate RAFT agent due to the specific coordination to zinc porphyrins; (ii) the continuous chain initiation in the presence of a small amount of TEOA^•^. The above control experiments suggested that our acrylamide polymers contain both TEOA^•^ and R groups (from the RAFT agent) as the polymer chain ends.

**Figure 4:**
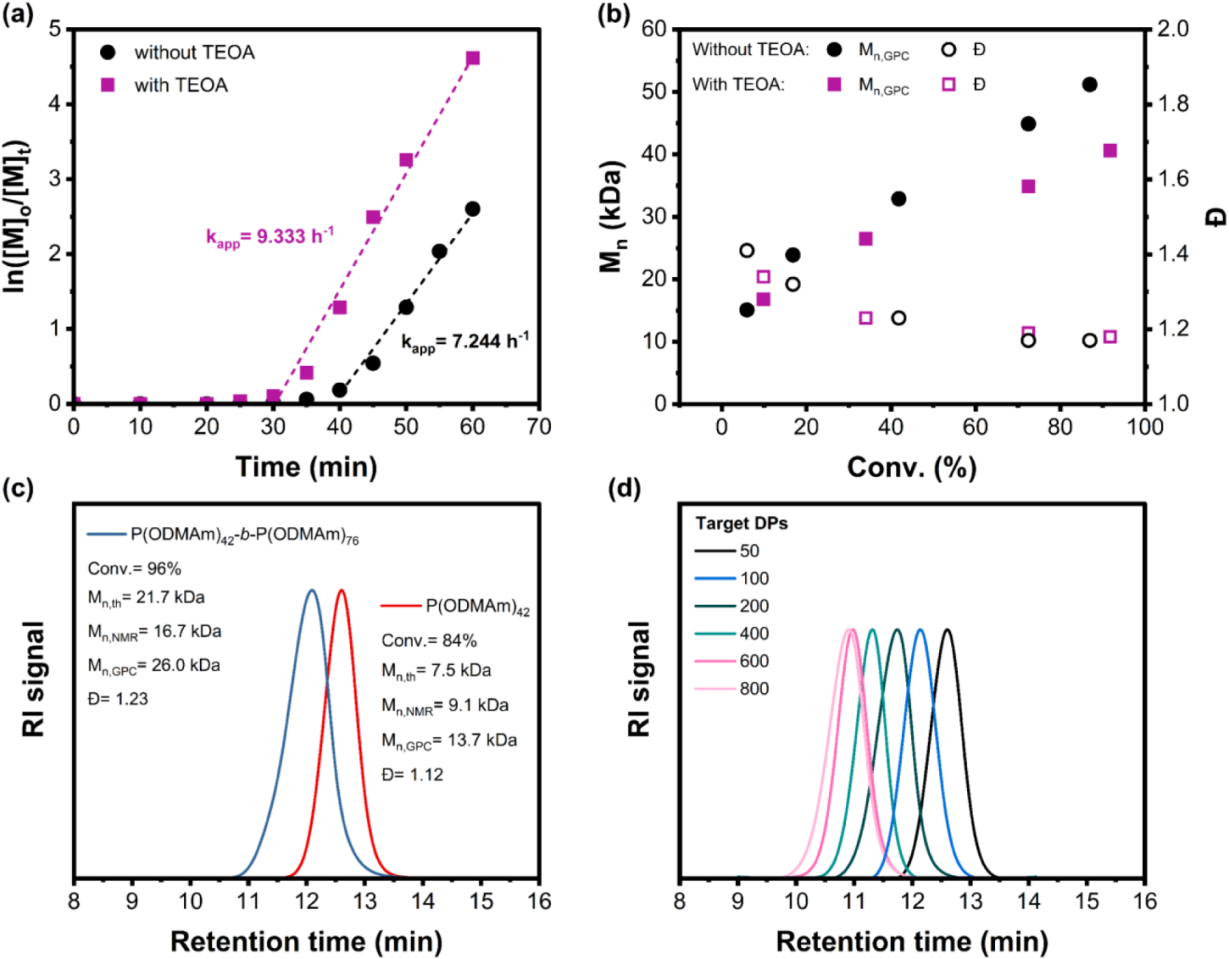
(a) Kinetics study of PET-RAFT polymerization of ODMAm in the absence of oxygen at room temperature with CBPA as the chain transfer agent, ZnTPS_4_ as the photocatalyst under red- light irradiation, using the ratio of [M]:[CTA]:[PC] = 200:1:0.01 and [PC]:[M] = 50 ppm in different solvent systems. (b) Evolution of *M*_n_ and Đ versus monomer conversion in the absence and presence of TEOA as a scarifying electron donor. (c) GPC traces of P­(ODMAm) macro-CTAs and their diblock copolymer synthesized via *in situ* chain extension in 70% H_2_O/DMSO. (d) GPC traces of P­(ODMAm) representing different degrees of polymerization synthesized via ZnTPS_4_-mediated PET-RAFT polymerization.

A chain-extension experiment was performed under optimized reaction conditions to investigate the livingness of the synthesized homopolymer. The synthesis of macro-CTA was carried out in 70% H_2_O/DMSO with a target DP of 50 under red-light irradiation for an hour, affording a polymer with 84% monomer conversion, an *M*_n_ of 13.7 kDa, and a Đ of 1.12, which was named as P­(ODMAm)_42_. The unpurified P­(ODMAm)_42_ macro-CTA was used for chain extension by adding additional ODMAm with a target DP of 50, the solution was purged with N_2_, and polymerized under the same conditions for an additional hour. The block ratio was determined as [M]:[macro-CTA] = 76:1 from ^1^H NMR spectroscopy before polymerization by using the same method as described above, and the resulting block copolymer was named as P­(ODMAm)_42_-*b*-P­(ODMAm)_76_. After 45 min of irradiation, a monomer conversion of 96% was achieved, as determined by ^1^H NMR spectroscopy. The significant decrease in the GPC retention time, corresponding to an *M*_n_ of 26.0 kDa and a Đ of 1.23, confirms the high end-group functionality (Figure 4c). In addition, polymerization with variable target DPs from 50 to 800 was carried out under red-light irradiation, and all polymers were able to achieve high monomer conversion within 1 h of irradiation (Table S9). The maximum control of polymer molecular weight was determined at a target DP of 600, with good agreement between GPC molecular weight and theoretical molecular weight (Figure S25), and no significant tailing was observed in the GPC traces (Figure 4d). At a target DP of 800, a slightly broader Đ of 1.24 was obtained.

Given the success in achieving controlled polymerization of poly­(*N*-oxide) through ZnTPS_4_-mediated PET-RAFT polymerization of ODMMAm and ODMAm under red-light irradiation, we further challenged our system by conducting polymerization over a range of monomer concentrations (0.25 to 3 M), and the results are summarized in Table 4. When the polymerization was performed at a 1.5 M monomer concentration with the same PC loading as the optimal condition mentioned above ([PC]/[M] = 50 ppm) under a degassed environment, however, no monomer conversion was achieved (Table 4, entry 2). Upon increasing the PC loading to 100 ppm, the polymerization proceeded efficiently, affording a polymer with a monomer conversion of 93% in 1 h, an *M*_n_ of 30.4 kDa, and a Đ of 1.18 (Table 4, entry 3). A similar polymerization efficacy was achieved at 0.75 M with the same PC loading of 100 ppm (Table 4, entry 4). Further diluting the polymerization mixture to 0.5 and 0.25 M required a gradual increase in PC loading to 200 and 300 ppm, respectively. After optimizing the catalyst loading, excellent control over polymerization was achieved at a 0.25 M monomer concentration, suggesting that our system has the potential to be applied in the synthesis of protein–polymer conjugates.

**Table 4:** Varying Monomer Concentration in PET-RAFT Polymerization of ODMAm^a^

Entry	[M]:[CBPA]:[ZnTPS_4_]:[TEOA]	[PC]/[M] (ppm)	[M] (M)	α (%)^b^	*M*_n,th_ ^c^ (kDa)	*M*_n,GPC_ ^d^ (kDa)	Đ^d^	Dev.^e^
1^f^	200:1:0.01:5	50	3	96	33.5	38.4	1.19	14%
2	200:1:0.01:5	50	1.5	0	---	---	---	---
3	200:1:0.02:5	100	1.5	93	32.3	30.4	1.18	6%
4	200:1:0.02:5	100	0.75	84	29.2	30.9	1.18	6%
5	200:1:0.04:5	200	0.5	86	29.3	33.3	1.18	12%
6	200:1:0.06:5	300	0.25	58	20.3	33.2	1.20	64%

a Polymerization was carried out under deoxygenated conditions with the initial ratio of [M]:[CBPA]:[TEOA] = 200:1:5 in 70% H_2_O/DMSO. The polymerization mixture was irradiated with red light for 1 h.

b Determined by ^1^H NMR spectroscopy.

c Theoretical molecular weight was calculated using the following equation: *M*_n,th_= [*M*_o_]/[CTA] × α × *M*_w,monomer_ + *M*_w,CTA_, where [*M*_o_], [CTA], α, *M*_w,monomer_, and *M*_w,CTA_ correspond to the initial monomer concentration, initial CTA concentration, monomer conversion determined by ^1^H NMR, molar mass of the monomer, and molar mass of the CTA.

d Molecular weight (*M*_n,GPC_) and dispersity (Đ) were determined by GPC analysis (TFE as eluent) calibrated using PMMA standards.

e Deviation (Dev.)= |*M*_n,th_ – *M*_n,GPC_|/*M*_n,th_ × 100%.

f Taken from entry 3 of Table 3 as the reference.

To demonstrate temporal control of PET-RAFT polymerization of ODMAm monomer, a light on/off experiment was performed under the optimal conditions by using CPDT as a RAFT agent in an open-to-air environment, as shown in Figure 5a. The polymerization process can be effectively paused and resumed by switching the light on and off. A negligible increase in monomer conversion during the dark period can be attributed to the presence of residual radical species. Polymerization can be reinitiated immediately by switching the light on, indicating the livingness of our polymerization system and the excellent responsiveness (Figure 5b). The polymer achieved after three repeated on/off cycles has an *M*_n_ of 60.5 kDa and a Đ of 1.20 at a monomer conversion of 96%.

**Figure 5:**
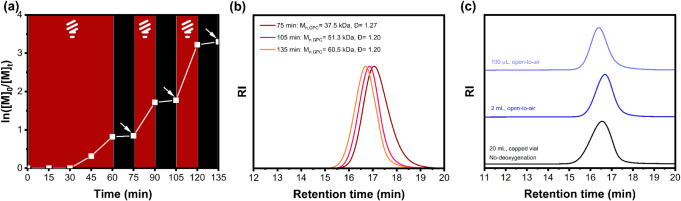
(a) Temporal control of ZnTPS_4_-mediated PET-RAFT polymerization of ODMAm under open-to-air conditions;. (b) GPC chromatogram of P­(ODMAm) at various time points as labeled in (a). (c) GPC chromatogram of P­(ODMAm) synthesized at various scales: 100 μL, 2 mL, and 20 mL.

Low-volume RDRP has demonstrated broad and practical applications, such as high-throughput screening,^72–76^ bioconjugation,^73,77^ and surface-initiated polymerization.^78^ However, the use of external deoxygenation methods, such as freeze–pump–thaw and purging with inert gas, on a small scale can lead to the loss of volatile substances. The oxygen-tolerant RDRP technique avoids the need for deoxygenation before polymerization, thus allowing polymer synthesis in a small volume. The versatility and effectiveness of our polymerization system were evaluated by conducting a series of polymerizations at 100 μL, 200 μL, 2 mL, 4 mL, 7 mL, and 20 mL, at the feed ratio of [M]:[CTA]:[ZnTPS_4_]:[TEOA] = 200:1:0.04:5 with a monomer concentration of 0.5 M in 70% H_2_O/DMSO under open-to-air conditions, by using acrylamide-based *N*-oxide (ODMAm) as a model monomer and CPDT as a RAFT agent. The polymerization results are summarized in Table S10. Despite the change in reaction volume, high monomer conversions (66–97%) were achieved in all of the reactions. The slightly higher dispersities can be attributed to the increased diffusion of oxygen into the system at low volume. However, negligible monomer conversion was achieved for the polymerization conducted on a 20 mL scale, even though the irradiation time had been prolonged to 2 h (entry 6, Table S10). This can be attributed to the large volume of dissolved oxygen in the solution, thus quenching the initiating radical species. Additional polymerization was performed on a 20 mL scale in a capped vial without using any external deoxygenation method, affording a polymer with an *M*_n_ of 73.5 kDa and a Đ of 1.26 (Figure 5c). These results indicate the great potential of the ZnTPS_4_ system for high-throughput screening and bioconjugation of poly­(*N*-oxide).

To demonstrate the versatility and effectiveness of ZnTPS_4_-mediated PET-RAFT system for *N*-oxide (meth)­acrylamide-based monomers, which is not only applicable to the *N*-oxide monomers mentioned above but also applicable to other hydrophilic monomers; we further expanded the monomer scope to other hydrophilic monomers. For instance, *N*-oxide monomers with different side chains (ODEMA, ODEMAm), methacrylate-based monomers with zwitterionic side chains, including sulfobetaine (DAPS) and phosphocholine (MPC), and other hydrophilic monomers (OEGMA_500_, DEMM-I) were polymerized at the fixed ratio of [M]:[CPADB]:[ZnTPS_4_]:[TEOA] = 200:1:0.01:5 in 70% H_2_O/DMSO (Table 5). Even though these methacrylate monomers, ODEMA, OEGMA_500_, and DEMM-I, achieved a lower monomer conversion in the range of 33–43% after 4 h of red-light irradiation, the obtained polymer still exhibits a narrow Đ; this can be attributed to the difference in monomer reactivity. Meanwhile, other zwitterionic monomers, DAPS, MPC, and ODEMAm, showed a high monomer conversion with excellent control over molecular weight and Đ. All polymerizations were performed in a controlled manner with low dispersity (Đ < 1.30) over a wide monomer scope (Figure S26), thus indicating the potential of the ZnTPS_4_-mediated PET-RAFT polymerization system in synthesizing functional polymers for various applications.

**Table 5:** Polymerization of Various *N*-Oxide and Zwitterionic monomers^a^

Entry	Monomer	α (%)^b^	*M*_n,th_ ^c^ (kDa)	*M*_n,GPC_ ^d^ (kDa)	Đ^d^	Dev.^e^
1	ODEMA	33	13.6	20.0	1.10	47%
2	ODEMAm	77	31.1	35.4	1.23	14%
3	DAPS	99	55.6	38.3	1.20	31%
4	MPC	97	57.6	63.5	1.14	10%
5	OEGMA_500_	43	42.9	38.3	1.19	11%
6	DEMM-I	35	23.2	20.7	1.25	11%

a Polymerization was carried out under deoxygenated conditions with the initial ratio of [M]:[CPADB]:[PC]:[TEOA] = 200:1:0.01:5, [PC]:[M] = 50 ppm, and [M] = 3 M in 70% H_2_O/DMSO. The polymerization mixture was irradiated with red light for 4 h.

b Determined by ^1^H NMR spectroscopy.

c Theoretical molecular weight was calculated using the following equation: *M*_n,th_= [*M*_0_]/[CTA] × α × *M*_w,monomer_ + *M*_w,CTA_, where [*M*_0_], [CTA], α, *M*_w,monomer_, and *M*_w,CTA_ correspond to the initial monomer concentration, initial CTA concentration, monomer conversion determined by ^1^H NMR, molar mass of the monomer, and molar mass of the CTA.

d Molecular weight (*M*_n,GPC_) and dispersity (Đ) were determined by GPC analysis (TFE as eluent) calibrated using PMMA standards.

e Deviation (Dev.)= |*M*_n,th_ – *M*_n,GPC_|/*M*_n,th_ × 100%.

The biocompatibility of *N*-oxide monomers and their corresponding polymers was evaluated by MTT assay for cell viability and Lactate Dehydrogenase (LDH) assay for cell apoptosis on the fibroblast cell line (L929 cells). The ODEMA monomer and its corresponding polymer were used as references, as they have demonstrated excellent biocompatibility and are widely reported for biological applications.^34,69^ The data are shown in Figure S27. Negligible cell death was observed in the MTT assay when L929 was exposed to the monomer solutions at varying concentrations up to 400 μg/mL (Figure S27a). The LDH assay further confirms that there is no cell apoptosis caused by our *N*-oxide monomers (Figure S27b). In addition, similar results were observed when the cells were exposed to our poly­(*N*-oxide) samples at concentrations up to 800 μg/mL (Figure S27c and S27d). These results suggest a great biocompatibility of *N*-oxide monomers and their polymers synthesized by ZnTPS_4_-mediated PET-RAFT polymerization, which allows for further use of this polymerization system for advanced biological applications.

## 3 Conclusions

We have addressed the challenges of producing a well-defined (meth)­acrylamide-based *N*-oxide polymer through a thermally initiated RAFT polymerization technique, due to unexpected side reactions between the monomer and the chain transfer agent. PET-RAFT polymerization was adapted to overcome the synthetic challenges of such monomers by using a water-soluble zinc­(II) porphyrin derivative under red-light irradiation. The synthesized polymers showed a narrow molecular weight distribution (Đ < 1.30) over a wide targeted degree of polymerization (25–800), despite the loading of photocatalyst at ppm levels. The retention of chain-end functionality was confirmed by a successful *in situ* chain extension, where acrylamide-based monomers were found to be superior to methacrylamide monomers. Polymerization can be conducted at various monomer concentrations (0.25–3 M) to afford polymers with narrow polymer dispersity. This method also enabled controlled polymerization of acrylamide-, methacrylate-based *N*-oxide, zwitterionic monomers (i.e., sulfobetaine, phosphocholine), cationic, and hydrophilic oligo­(ethylene glycol) monomers. This work greatly expands the scope of monomers that can be polymerized by the PET-RAFT technique under red-light irradiation.

## Supplementary Material



## Notes

Experimental details, photoreaction setup, additional characterization data, kinetic studies, and GPC chromatograms (PDF)
