# Supplementary material for: Red-Light-Induced PET-RAFT Polymerization to Afford (Meth)acrylamide-Based Poly(N‑oxide) and Other Hydrophilic Polymers Featuring Neutral, Cationic, and Zwitterionic Groups as Solubilizing Side Chains
Source: Macromolecules. 2026 Feb 13;59(4):2412–25. doi: 10.1021/acs.macromol.5c03186 (PMC12947689; doi:10.1021/acs.macromol.5c03186)
Supplement: Supplementary file 1 [file ma5c03186_si_001.pdf]

SUPPORTING INFORMATION FOR

Red-Light-Induced PET-RAFT Polymerization to Afford (Meth)acrylamide-based Poly(*N*-oxide) and Other Hydrophilic Polymers Featuring Neutral, Cationic and Zwitterionic Groups as Solubilizing Side Chains

Van-Sieu Luc[a],[b],[c], Kien-Sam Banh[a], Thach-Thao T. Nguyen[a], Min-Hsuan Hsieh[d], Tung-Kung Wu[e],[h], Vitalijus Karabanovas[f], Ricardas Rotomskis[f], Simona Steponkiene[f], Yaw-Kuen Li[a],[h], Ying-Nien Chou[g], I-Chi Lee[d] and Chia-Chih Chang[a],[h]*

[a] Department of Applied Chemistry, National Yang Ming Chiao Tung University, Hsinchu 300093, Taiwan

[b] Institute of Chemistry, Academia Sinica, Taipei 11529, Taiwan

[c] Sustainable Chemical Science and Technology (SCST), Taiwan International Graduate Program (TIGP), Academia Sinica, Taipei 11529, Taiwan

[d] Department of Biomedical Engineering and Environmental Sciences, National Tsing Hua University, Hsinchu, 300044 Taiwan

[e] Department of Biological Science and Technology, National Yang Ming Chiao Tung University, Hsinchu 300093, Taiwan

[f] Biomedical Physics Laboratory, National Cancer Institute, Vilnius LT-08406, Lithuania

[g] Department of Chemical Engineering, National Chung Cheng University, Minhsiung, Chiayi 62102, Taiwan

[h] Center for Emergent Functional Matter Science, National Yang Ming Chiao Tung University, Hsinchu 300093, Taiwan

*Chia-Chih Chang: cchang113ac@nycu.edu.tw

Table of Contents

1. Materials and Methods 1

1.1. Materials 1

1.2. General instrumentation 1

1.3. Photopolymerization Setup 2

2. Experimental Section 2

2.1. Synthesis of zinc(II) *meso*-tetra (4-sulfonatophenyl) porphyrin tetrasodium ($ZnTPS_4$) ... 2

2.2. Synthesis of *N*-2-(diethylamino)ethyl methacrylamide (DEAMA) 3

2.3. General procedure to synthesize *N*-oxide monomer 3

2.4. General procedure for the synthesis of P(ODMMAm) via thermally-initiated RAFT polymerization 4

2.5. General procedure for the synthesis of P(ODMMAm) via ZnTPP-mediated PET-RAFT polymerization 4

2.6. General procedure for the synthesis of P(ODMMAm) via $ZnTPS_4$-mediated PET-RAFT polymerization 4

2.7. Kinetics studies of P(ODMMAm) synthesized via PET-RAFT in 30% H_2O/DMSO....... 5

2.8. Kinetics studies of P(ODMMAm) synthesized via PET-RAFT in 50% H_2O/DMSO....... 5

2.9. Kinetics studies of P(ODMMAm) synthesized via PET-RAFT in 70% H_2O/DMSO....... 5

2.10. Synthesis of P(ODMMAm) homopolymers with various DPs 6

2.11. *In situ* chain extension of P(ODMMAm) and P(ODMAm) 6

2.12. Temporal control 6

2.13. MTT assay (Cell Viability Assessment) 7

2.14. LDH assay for Cytotoxicity 7

3. Supporting tables and figures 9

4. Characterization details of the synthesized compounds 28

5. Reference 32

1. Materials and Methods

1.1. Materials

5,10,15,20-tetrakisphenylphorphyrin (TPP, 97%, Macklin), zinc acetate $(Zn(OAc)_2$, 99%, Sigma-Aldrich), *N*-[3-(dimethylamino)propyl]methacrylamide (DMAPMA, 99%, Macklin), triethanolamine (TEOA, 99%, Sigma-Aldrich), *N*-[3-(dimethylamino)propyl]acrylamide (DMAPA, 98%, Macklin), *N,N*-diethylethylenediamine (DEA, 99%, Macklin), methacryloyl chloride (MACl, 95%, Macklin), acryloyl chloride (ACl, 98%, Macklin), triethylamine (TEA, 98%, J.T. Baker), triethanolamine (TEOA, > 99%, Sigma-Aldrich), 4,4'-Azobis(4-cyanovaleric acid) (ACVA, 98%, Sigma-Aldrich), 2,2'-Azobis[2-(2-imidazolin-2-yl)propane]dihydrochloride (VA-044, 98% Maxtrix Scientific), *meta*-chloroperoxybenzoic acid (*m*CPBA, 70-75%, Acros Organics), 4-cyano-4-(thiobenzoylthio)pentanoic acid (CPADB, 97%, Sigma-Aldrich), 2-(((butylsulfanyl)carbothioyl)sulfanyl)propanoic acid (BTPA, 95%, TCI), 3-((((1-carboxyethyl)thio)carbonothioyl)thio)propanoic acid (TTCP, 95%, TCI) were directly used without further purification. Deionized water (DI H_2O, 18.2 MΩ·cm, Sartorius Arium Pro) was used. 4-cyano-4-(((decylthio)carbonothioyl)thio)pentanoic acid (CPDT) and 4-cyano-4-(((butylthio)carbonothioyl)thio)pentanoic acid (CBPA) were synthesized in accordance with the literature[1], except 1-decanethiol and 1-butanethiol were used instead of 1-dodecanethiol. Zinc *meso*-tertraphenyl porphyrin (ZnTPP) and 5,10,15,20-Tetrakis(4-sulfonatophenyl) porphyrin tetrasodium (TPS_4) were prepared according to the literature, and the chemical structures were confirmed by ^1H NMR.[2, 3] All reaction solvents were purchased from commercial vendors and directly used without further purification.

1.2. General instrumentation

Nuclear Magnetic Resonance (NMR): ^1H NMR and ^{13}C NMR spectra were obtained on Agilent 400-MR DDR2 400 MHz spectrometer, JEOL JNM-ECZS 400 MHz spectrometer (400 MHz for ^1H spectra; 100 MHz for ^{13}C spectra) or Agilent VNMRS 600 spectrometer (600 MHz for ^1H spectra; 150 MHz for ^{13}C spectra) with $CDCl_3$, DMSO-d_6 or D_2O as solvents. The chemical shifts are given in parts per million (ppm) using the solvent peak, such as chloroform-d (δ_H= 7.26 ppm; δ_C= 77.16 ppm), DMSO-d_6 (δ_H= 2.5 ppm; δ_C= 39.52 ppm), or D_2O (δ_H= 4.79 ppm) as an internal standard.

Mass Spectrometry (MS): Electrospray ionization (ESI) mass spectra were acquired on an Impact HD Q-ToF mass spectrometry (Bruker, Germany) at the Center for Advanced Instrumentation of National Yang Ming Chiao Tung University.

Gel Permeation Chromatography (GPC): The crude polymerization mixtures were analyzed directly by gel permeation chromatography using TFE as eluents. Polymer sample solutions were prepared in the GPC eluent and filtered through a PTFE filter (0.45 μm) before injection.

TFE-GPC was performed using a Tosoh EcoSEC HLC-8320GPC system equipped with a guard column TSKgel SuperAW-H (Tosoh) and 3 SuperAWM-H columns (Tosoh) in series. The 2,2,2-trifluoroethanol (TFE) containing 20 mM sodium trifluoroacetate was used as a mobile phase at a flow rate of 0.6 mL/min or 0.4 mL/min. The column temperature was maintained at 40°C. A molecular weight calibration curve was produced using commercial narrow molecular weight distribution poly(methyl methacrylate) (PMMA) standards with molecular weights ranging from 965,000 to 2,500.

UV-Vis spectrometry: A UV-Vis spectrum was recorded by using an ultraviolet-visible-near infrared spectrometer (Jasco V-770) with a quartz cuvette cell (10 mm light path) using DI H_2O or DMSO as solvents.

1.3. Photopolymerization Setup

Photopolymerization was carried out in a 7 mL scintillation vial sealed with a rubber septum. The reaction mixtures were degassed before irradiation with an LED light bulb (PAR38 18 W, VitaLED Technologies, Taiwan) with the irradiation wavelength λ_{max}= 625 nm. The vial was placed 10 cm away from the light bulb. The temperature was monitored and remained around 25°C throughout the experiment by using a cooling fan.

2. Experimental Section

2.1. Synthesis of zinc(II) *meso*-tetra (4-sulfonatophenyl) porphyrin tetrasodium ($ZnTPS_4$)

To a stirred solution of TPS_4 (0.3 g, 0.29 mmol, 1 equiv.) in DI H_2O (10 mL) at 100°C, a solution of $Zn(OAc)_2$ (0.27 g, 1.45 mmol, 5 equiv.) in DI H_2O (10 mL) was added via syringe and the mixture was heated at 100°C for 4 h. The progress of the reaction was monitored by UV-Vis spectroscopy. After noticing the absence of free porphyrin absorption peaks from the UV-Vis spectrum, the solution was

cooled down to room temperature and directly loaded onto flash column chromatography (reverse phase C_{18} silica gel, 0-50% MeOH/H_2O). The product fractions were combined, and most of the MeOH was evaporated under reduced pressure. The aqueous solution was lyophilized, affording the $ZnTPS_4$ as a spongy brown powder (0.22 g, 69%). ^{1}H NMR (600 *MHz*, DMSO-d_6): 8.76 (*s*, 8H, pyrazole-H), 8.10 (*m*, 8H, Ph-CH), 7.98 (*m*, 8H, Ph-CH). UV-Vis (λ_{max} in H_2O, 25°C): 420, 556, 593 nm.

2.2. Synthesis of *N*-2-(diethylamino)ethyl methacrylamide (DEAMA)

To an ice-cooled solution of DEA (7.2 mL, mmol, 1 equiv.) in THF (40 mL), MACl (5.3 mL, mmol, 1 equiv.) was added dropwise over 15 minutes. Upon addition, the mixture was allowed to stir overnight at room temperature. The white precipitate was removed by filtration and washed with THF. The filtrate was evaporated to dryness prior to purification by flash column chromatography (SiO_2, 50% EA/Hex), affording a yellowish viscous oil (4.5 g, 49%). ^{1}H NMR (400 *MHz*, $CDCl_3$): 6.60 (br, 1H), 5.69 (s, 1H), 5.27 (s, 1H), 3.31 (q, *J*= 5.6, 5.0 *Hz*, 2H) 2.60-2.43 (m, 6H), 1.93 (s, 3H), 0.99 (t, *J*= 7.2 *Hz*, 6H). ^{13}C (100 *MHz*, $CDCl_3$): 168.3, 140.0, 119.5, 51.3, 46.9, 37.0, 18.7, 12.1.

2.3. General procedure to synthesize *N*-oxide monomer

To an ice-cooled solution of DMAPMA (12 g, 70.5 mmol, 1 equiv.) in DCM (400 mL), *m*CPBA (20.4 g, 118.2 mmol, 1.5 equiv.) was added portionwise over 30 mins. Upon addition, the mixture was warmed up to room temperature and stirred for 4 h. The solvent was evaporated under reduced pressure, and the crude oil was purified by flash column chromatography (neutral Al_2O_3, 0% to 20% MeOH/DCM). The sample was dried overnight under vacuum, affording *N*-oxide-3-(*N,N*-dimethylamino)propyl methacrylamide (ODMMAm) as a white powder (12.0 g, 91%). ^{1}H NMR (600 MHz, $CDCl_3$): 8.80 (br, 1H), 5.73 (s, 1H), 5.23 (s, 1H), 3.40-3.31 (m, 4H), 3.15 (s, 6H), 2.15-2.02 (q, 2H), 1.89 (s, 3H). ^{13}C NMR (150 MHz, $CDCl_3$): 168.7, 139.9, 119.8, 69.5, 59.3, 37.9, 23.6, 18.8. HR-ESI MS ($C_9H_{18}N_2O_2$): cald. 186.1368, found 187.1504 $[M+H]^+$.

Other (metha)acrylamide and (metha)acrylate monomers were prepared according to a similar procedure. The characterization data were described as follows:

N-oxide-2-(*N,N*-diethylamino)ethyl methacrylamide (ODEMAm): white powder, 80% yield. ^{1}H NMR (600 *MHz*, $CDCl_3$): 9.48 (br, 1H), 5.74 (s, 1H), 5.22 (s, 1H), 3.72-3.62 (m, 2H), 3.34-3.26 (m, 2H), 3.23-3.16 (m, 4H), 1.86 (s, 3H), 1.21 (t, *J*= 7.3 Hz, 6H) ^{13}C NMR (150 *MHz*, $CDCl_3$): 168.3, 139.2,

120.4, 62.4, 60.0, 35.5, 18.5, 8.8. HR-ESI MS ($C_{10}H_{20}N_2O_2$): cald. 200.1525, found 201.1605 $[M+H]^+$.

N-oxide-3-(*N,N*-dimethylamino)propyl acrylamide (ODMAm): off-white powder, 90% yield. ^{1}H NMR (600 *MHz*, $CDCl_3$): 9.04 (br, 1H), 6.48-5.98 (m, 2H), 5.54 (dd, *J*= 9.5, 2.5 Hz, 1H), 3.45-3.38 (m, 2H), 3.36 (t, *J*= 6.8 Hz, 2H), 3.17 (s, 6H), 2.12 (p, *J*= 6.7 Hz, 2H). ^{13}C (150 *MHz*, $CDCl_3$): 166.3, 131.6, 125.5, 69.3, 59.4, 37.4, 23.7. HR-ESI MS ($C_8H_{16}N_2O_2$): cald. 172.1212, found 173.1290 $[M+H]^+$.

2.4. General procedure for the synthesis of P(ODMMAm) via thermally-initiated RAFT polymerization

To a 7 mL scintillation vial equipped with a magnetic stir bar, ODMMAm (0.56 g, 3.0 mmol, 200 equiv.), CPADB (35 μL of 143 mM stock solution in DMSO, 0.015 mmol, 1 equiv.), and ACVA (14 μL of 36 mM solution in DMSO, 1.5 μmol, 0.1 equiv.), and 0.62 mL of DMSO were added. The vial was sealed with a rubber septum and degassed by bubbling N_2 for at least 30 minutes. Polymerization was conducted in an oil bath at 70°C for 12h. The reaction mixture was then analyzed by ^{1}H NMR (D_2O) and GPC (TFE) to measure the conversion, number-average molecular weight (M_n), and molecular weight distribution (M_w/M_n).

2.5. General procedure for the synthesis of P(ODMMAm) via ZnTPP-mediated PET-RAFT polymerization

To a 7 mL scintillation vial equipped with a magnetic stir bar, ODMMAm (0.56 g, 3.0 mmol, 200 equiv.), CPADB (52 μL of 0.286 M stock solution in DMSO, 0.015 mmol), ZnTPP (163 μL of 1.47 mM stock solution in DMSO, 0.15 μmol), and 846 μL DMSO were added. The vial was sealed with a rubber septum and degassed by bubbling N_2 for 20 minutes. The reaction vial was placed in a custom-built photoreactor and stirred at 400 rpm for 4h. An aliquot was taken out at a determined time point and analyzed by ^{1}H NMR (D_2O) and GPC (TFE) to measure the monomer conversion, number-average molecular weight (M_n), and molecular weight distribution (M_w/M_n).

2.6. General procedure for the synthesis of P(ODMMAm) via ZnTPS$_4$-mediated PET-RAFT polymerization

To a 7 mL scintillation vial equipped with a magnetic stir bar, A-ODMA (0.56 g, 3.0 mmol, 200 equiv.), CPADB (52 μL of 0.286 M stock solution in DMSO, 0.015 mmol), ZnTPPS (163 μL of 0.92 mM stock

solution in H_2O, 0.15 μmol), 0.54 mL H_2O, and 0.25 mL DMSO were added. The vial was sealed with a rubber septum and degassed by bubbling N_2 for 30 minutes. The reaction vial was placed a custom-built photoreactor and stirred at 400 rpm for 4h. An aliquot was taken out at a determined time point and analyzed by ^1H NMR (D_2O) and GPC (TFE) to measure the monomer conversion, number-average molecular weight (M_n), and molecular weight distribution (M_w/M_n).

2.7. Kinetics studies of P(ODMMAm) synthesized via PET-RAFT in 30% H_2O/DMSO

To a 7 mL scintillation vial equipped with a magnetic stir bar, A-ODMA (1.12 g, 6.0 mmol, 200 equiv.), CPADB (105 μL of 0.286 M stock solution in DMSO, 0.03 mmol), ZnTPP (203 μL of 1.47 mM stock solution in DMSO, 0.3 μmol), 0.6 mL H_2O, and 0.2 mL DMSO were added. The vial was sealed with a rubber septum and degassed by bubbling N_2 for 30 minutes. The reaction vial was placed in a custom-built photoreactor and stirred at 400 rpm for 4h. An aliquot was taken out at a determined time point under N_2 atmosphere and analyzed by ^1H NMR (D_2O) and GPC (TFE) to measure the monomer conversion, number-average molecular weight (M_n), and molecular weight distribution (Đ).

2.8. Kinetics studies of P(ODMMAm) synthesized via PET-RAFT in 50% H_2O/DMSO

To a 7 mL scintillation vial equipped with a magnetic stir bar, A-ODMA (1.12 g, 6.0 mmol, 200 equiv.), CPADB (105 μL of 0.286 M stock solution in DMSO, 0.03 mmol), $ZnTPS_4$ (326 μL of 0.92 mM stock solution in H_2O, 0.3 μmol), 674 μL H_2O, and 783 μL DMSO were added. The vial was sealed with a rubber septum and degassed by bubbling N_2 for 30 minutes. The reaction vial was placed a custom-built photoreactor and stirred at 400 rpm for 4h. An aliquot was taken out at a determined time point and analyzed by ^1H NMR (D_2O) and GPC (TFE) to measure the monomer conversion, number-average molecular weight (M_n), and molecular weight distribution (Đ).

2.9. Kinetics studies of P(ODMMAm) synthesized via PET-RAFT in 70% H_2O/DMSO

To a 7 mL scintillation vial equipped with a magnetic stir bar, A-ODMA (1.12 g, 6.0 mmol, 200 equiv.), CPADB (105 μL of 0.286 M stock solution in DMSO, 0.03 mmol), $ZnTPS_4$ (326 μL of 0.92 mM stock solution in H_2O, 0.3 μmol), 1074 μL H_2O, and 383 μL DMSO were added. The vial was sealed with a rubber septum and degassed by bubbling N_2 for 30 minutes. The reaction vial was placed a custom-built photoreactor and stirred at 400 rpm for 4h. An aliquot was taken out at a determined time point and analyzed by ^1H NMR (D_2O) and GPC (TFE) to measure the monomer conversion, number-average

molecular weight (M_n), and molecular weight distribution (Đ).

The kinetic studies of ODMAm were conducted in the same procedure as described above, in which the ODMAm monomer and CBPA chain transfer agent were used instead of ODMMAm and CPADB.

2.10. Synthesis of P(ODMMAm) homopolymers with various DPs

To a 7 mL vial equipped with a magnetic stir bar, 4.2 mg of CPADB (0.015 mmol, 1 equiv.), and 11.2 mg of TEOA (0.075 mmol, 5 equiv.) were inserted and dissolved in a mixture of 532 μL H_2O and 192 μL DMSO. Subsequently, various amounts of ODMMAm monomer and $ZnTPS_4$ photocatalyst (163 μL of 0.92 mM stock solution in H_2O, 0.15 μmol, 0.01 equiv.) were added to the vial. The reaction vial was placed in a custom-built photoreactor and stirred at 400 rpm for 4h. An aliquot was taken out at a determined time point and analyzed by ^{1}H NMR (D_2O) and GPC (TFE) to measure the monomer conversion, number-average molecular weight (M_n), and molecular weight distribution (Đ).

The polymerization of P(ODMAm) with various DPs was conducted in the same procedure as described above, in which ODMAm was used instead of ODMMAm.

2.11. *In situ* chain extension of P(ODMMAm) and P(ODMAm)

Chain extensions were performed using the same procedures as the polymerizations described above, employing $P(ODMMAm)_{50}$ synthesized as a macroinitiator under the same conditions and used directly for chain extension without further purification. All crude polymers were directly used in analysis by ^{1}H NMR and GPC without purification.

The chain-extension experiments of acrylamide-based *N*-oxide (ODMAm) were conducted in the same procedure as described above, in which ODMAm was used instead of ODMMAm.

2.12. Temporal control

To a 20 mL vial equipped with a magnetic stir bar, 16.9 mg of CPDT (0.045 mmol, 1 equiv.) and 2.58 g of ODMAm monomer (15 mmol, 200 equiv.) were dissolved in a mixture of 1443 μL H_2O and 689 μL DMSO under gentle stirring (200 rpm) in dark. Subsequently, a stock solution of ZnTPS4 (0.49 mL of 0.92 mM in H_2O, 4.5 μmol, 0.01 equiv.) and TEOA (0.17 mL of 1.34 mM in H_2O, 0.225 mmol, 5 equiv.) were added to the above solution. Upon complete mixing, 7 mL of the mixture was transferred to the 7 mL glass vial equipped with a magnetic stir bar and irradiated for 135 mins under red light

LEDs (625 nm, 35 mW/cm^2). At different time intervals (0, 15, 30, 45, 60, 75, 90, 105, 120, and 135 min), aliquots were taken and analyzed by ^{1}H NMR spectroscopy using D_2O as solvent. The GPC analysis was performed on the samples at 75, 105, and 135 min.

2.13. MTT assay (Cell Viability Assessment)

Cell viability was assessed using an MTT assay following the principles of ISO 10993 for in vitro biocompatibility evaluation. Three polymers, P(ODMMAm), P(ODMAm), and OPDEA, as well as their corresponding monomers (ODMMAm, ODMAm, and ODEA), were individually evaluated. Each polymer or monomer was directly dissolved in complete culture medium to obtain a concentration series of 50, 100, 200, 400, and 800 μg mL^{-1}. L929 fibroblast cells were seeded onto tissue culture polystyrene (TCPS) 96-well plates at a density of 1×10^4 cells per well and cultured under standard conditions (37 °C, 5% CO_2). Cells cultured on TCPS in polymer- and monomer-free medium were used as a positive control, while cells treated with Triton X-100 served as the negative control. After cell attachment, the culture medium was replaced with medium containing the designated polymer or monomer at the indicated concentrations and incubated for 24 h. Following incubation, the treatment media were removed, and MTT reagent (3-(4,5-dimethylthiazol-2-yl)-2,5-diphenyltetrazolium bromide) was added to each well. The plates were incubated at 37 °C for 2 h to allow the formation of formazan crystals. The MTT solution was then discarded, and dimethyl sulfoxide (DMSO) was added to dissolve the formazan products. Absorbance was measured at 570 nm using a microplate reader. Cell viability was normalized to the positive control group, which was defined as 1, to evaluate the concentration-dependent biocompatibility of the polymers and monomers.

2.14. LDH assay for Cytotoxicity

Cytotoxicity was further examined by quantifying lactate dehydrogenase (LDH) release using a commercial LDH detection kit (abcam, ab65393). L929 cells were treated with P(ODMMAm), P(ODMAm), OPDEA, or their corresponding monomers at concentrations of 50–800 μg mL^{-1}, following the same culture conditions and incubation period as described for the MTT assay. After 24 h of exposure, 100 μL of the culture supernatant from each well was collected and incubated with the LDH reaction mixture according to the manufacturer's instructions. The resulting colorimetric signal was measured using a microplate reader at 490 nm, with a reference wavelength of 630 nm. LDH release was used as an indicator of membrane damage and cytotoxic response, allowing assessment of the

concentration-dependent cytotoxicity of the three polymers and three monomers.

3. Supporting tables and figures

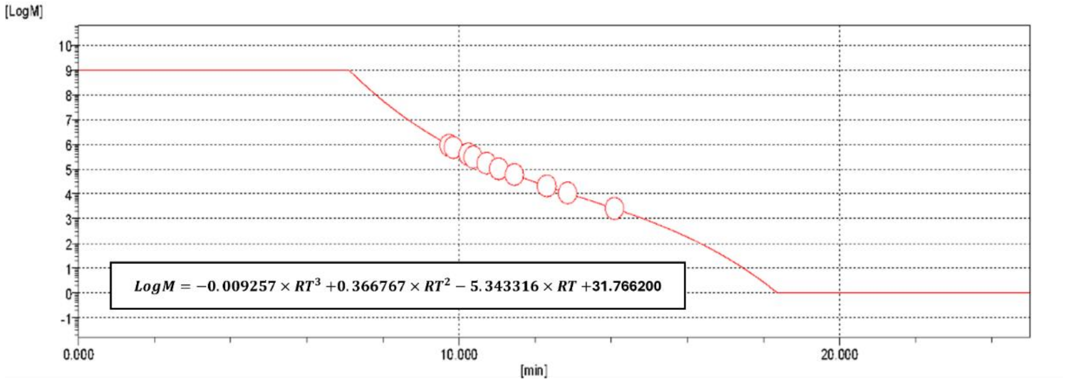

Figure S1. The calibration curve of narrow PMMA standards on the EcoSec HLC-8320GPC system (TFE + 20 mM CF_3COONa as eluent) at a flow rate of **0.6 mL/min**.

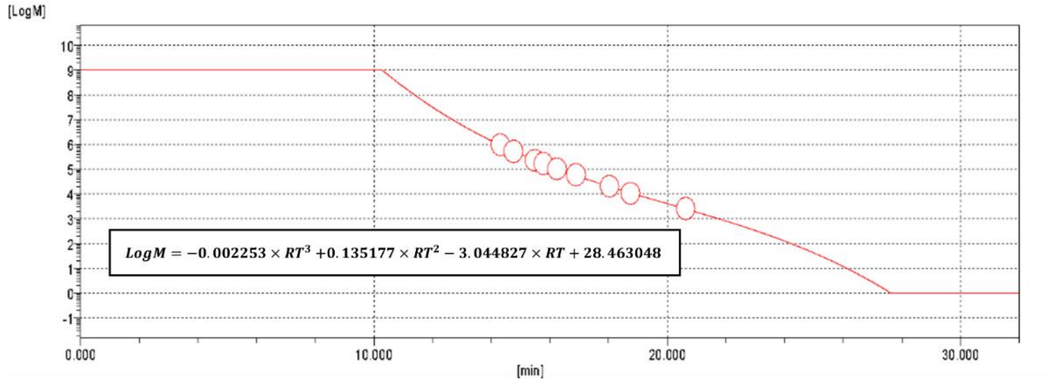

Figure S2. The calibration curve of narrow PMMA standards on the EcoSec HLC-8320GPC system (TFE + 20 mM CF_3COONa as eluent) at a flow rate of **0.4 mL/min**.

Table S1. List of PMMA standards retention time versus molecular weight at different flow rates

Flow rate (mL/min)	PMMA Molecular weight (Da)	RT (min)	Flow rate (mL/min)	PMMA Molecular weight (Da)	RT (min)
0.6	898,000	9.743	0.4	965,000	14.318
	740,000	9.853		505,000	14.762
	385,000	10.243		224,000	15.482
	296,000	10.375		166,000	15.775
	166,000	10.723		102,500	16.243
	102,500	11.045		58,700	16.897
	58,700	11.460		20,100	18.042
	20,100	12.307		10,500	18.748
	10,500	12.858		2,500	20.633
	2,500	14.088			

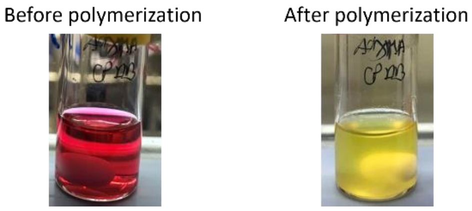

Figure S3. Digital images of crude polymerization mixture before and after reaction.

Table S2. Polymerization of ODMMAm through the conventional RAFT method[a]

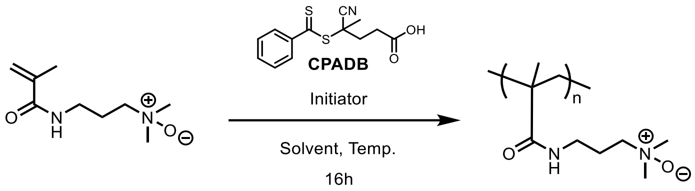

Entry	Initiator	Solvent	Conv. (%)[b]	$M_{n,th}$ (kDa)[c]	$M_{n,GPC}$ (kDa)[d]	Đ[d]	Dev. (%)[e]
1	ACVA	DMSO	13	5.1	28.2	1.80	453
2	VA-044		0	---	---	---	---
3	ACVA	H_2O	80	30.1	31.9	3.17	6
4	VA-044		70	26.3	28.9	1.54	10

[a]Polymerization was carried out under deoxygenated conditions with the initial ratio of [M]:[CTA]:[I]= 200:1:0.1 and [M]= 1.5 M. The polymerization temperature was set at 70°C for ACVA or 45°C for VA-044 as initiator; [b]Determined by ^{1}H NMR in D_2O; [c]Theoretical molecular weight was calculated using the following equation: $M_{n,th}$= $[M_o]/[CTA] \times conv. \times M_{w,monomer} + M_{w,RAFT}$, where $[M_o]$, [CTA], conv., $M_{w,monomer}$, and $M_{w,RAFT}$ correspond to initial monomer concentration, initial RAFT agent concentration, monomer conversion determined by ^{1}H NMR, molar mass of monomer, and molar mass of RAFT agent; [d]Molecular weight ($M_{n,GPC}$) and dispersity (Đ) were determined by GPC analysis (TFE as eluent) calibrated using PMMA standards

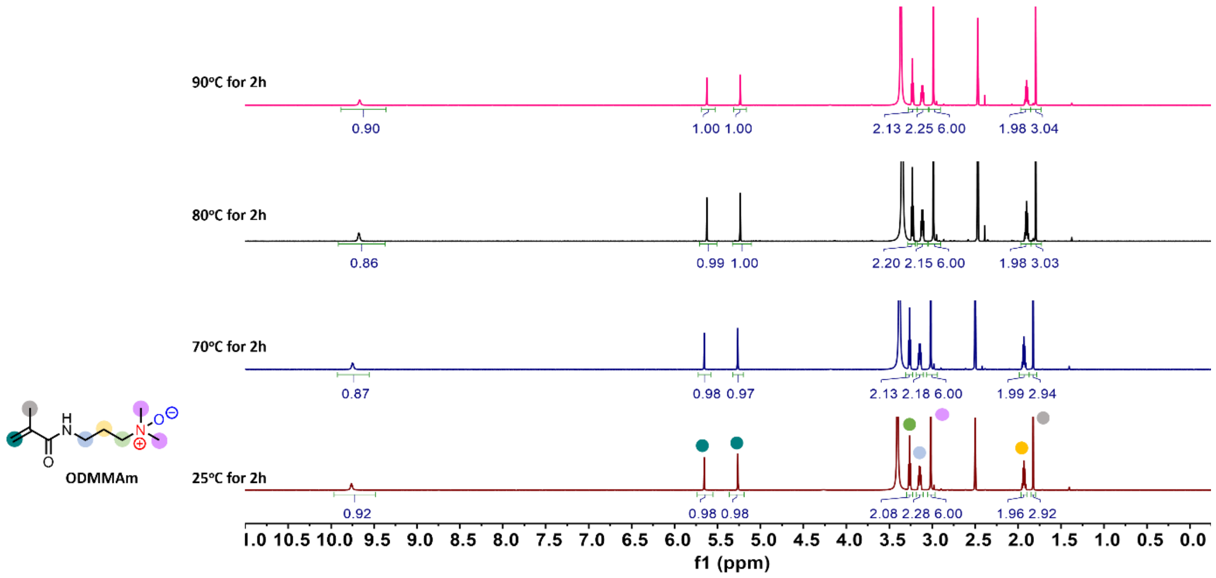

Figure S4. Stacked ^1H NMR (600 *MHz*, DMSO-d_6) of ODMMAm heating at various temperatures for 2 h.

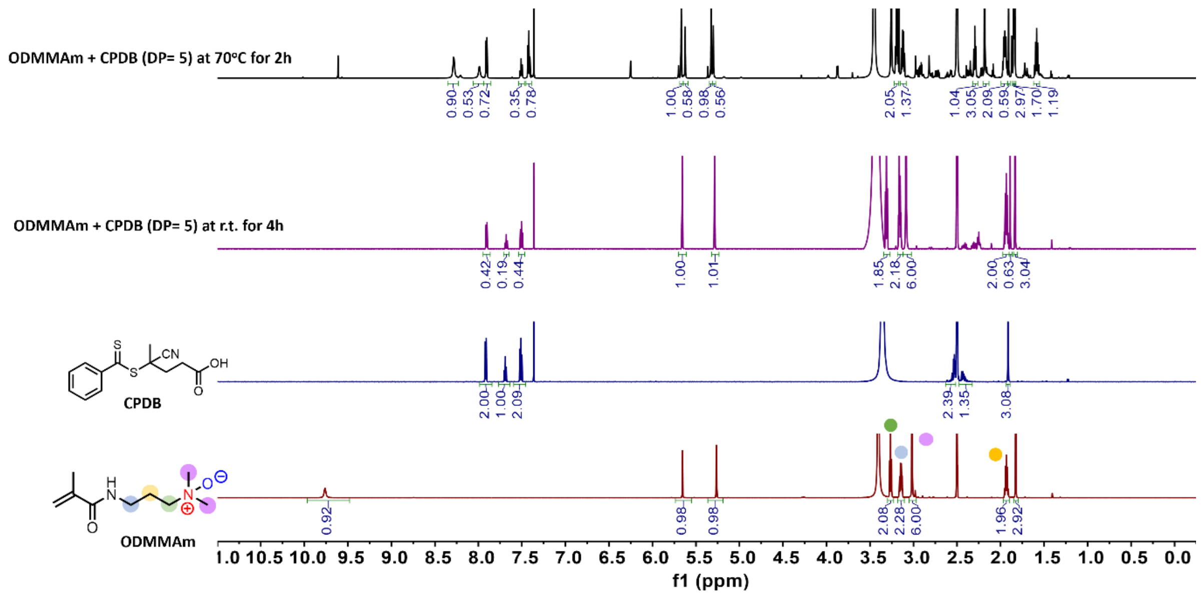

Figure S5. Stacked ^1H NMR (600 *MHz*, DMSO-d_6) of ODMMAm and CPADB upon heating at 70°C in the absence of radical initiator.

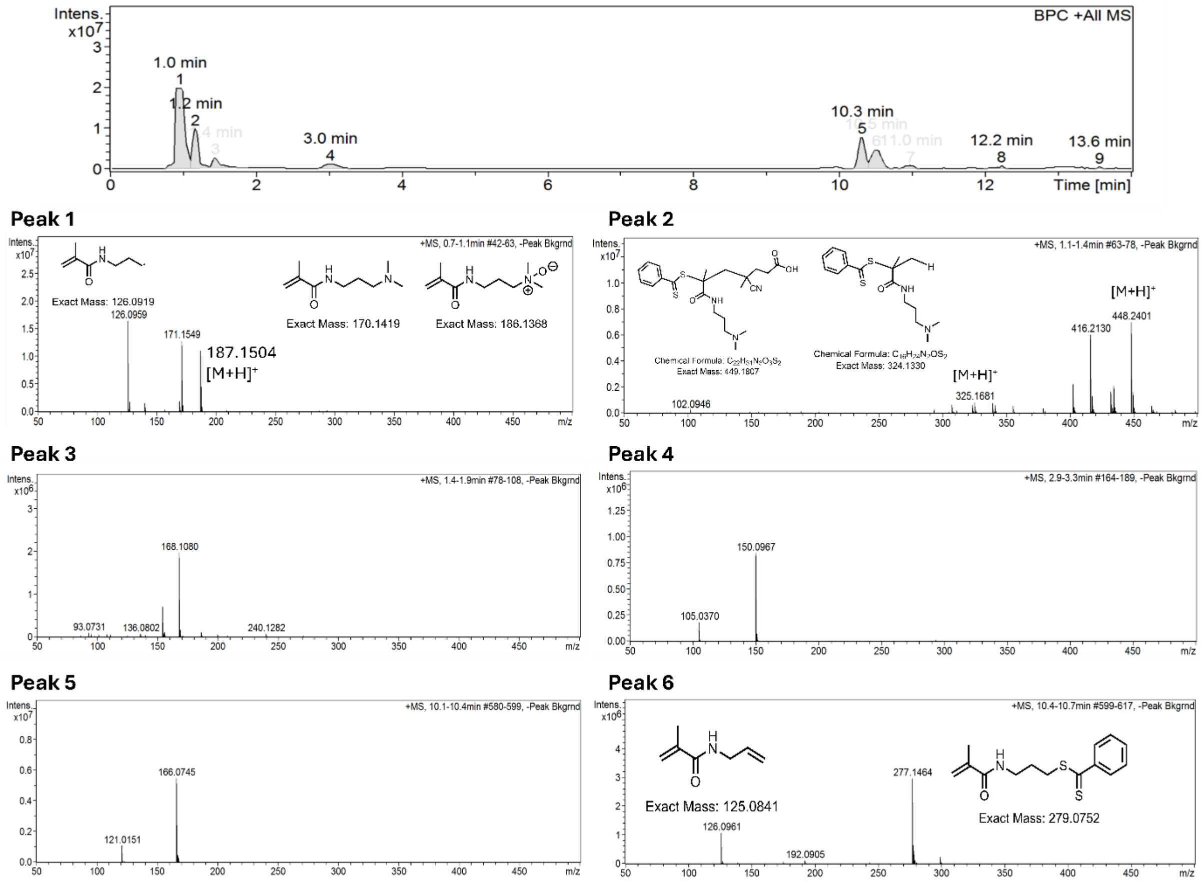

Figure S6. HPLC chromatogram of a crude reaction mixture between ODMMAm and CPADB at 70°C (top), the corresponding high-resolution mass spectroscopy of identified peaks (bottom).

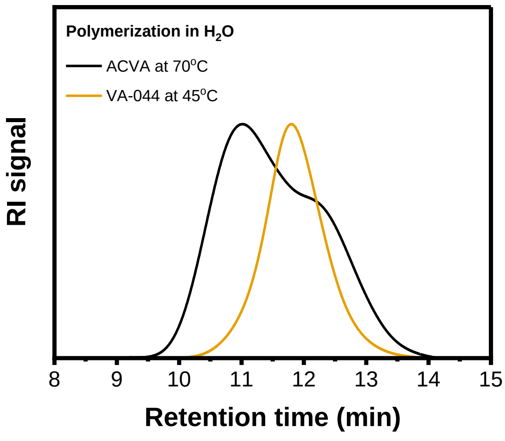

Figure S7. GPC traces of GPC traces of P(ODMMAm) initiated by ACVA and VA-044 in aqueous solution

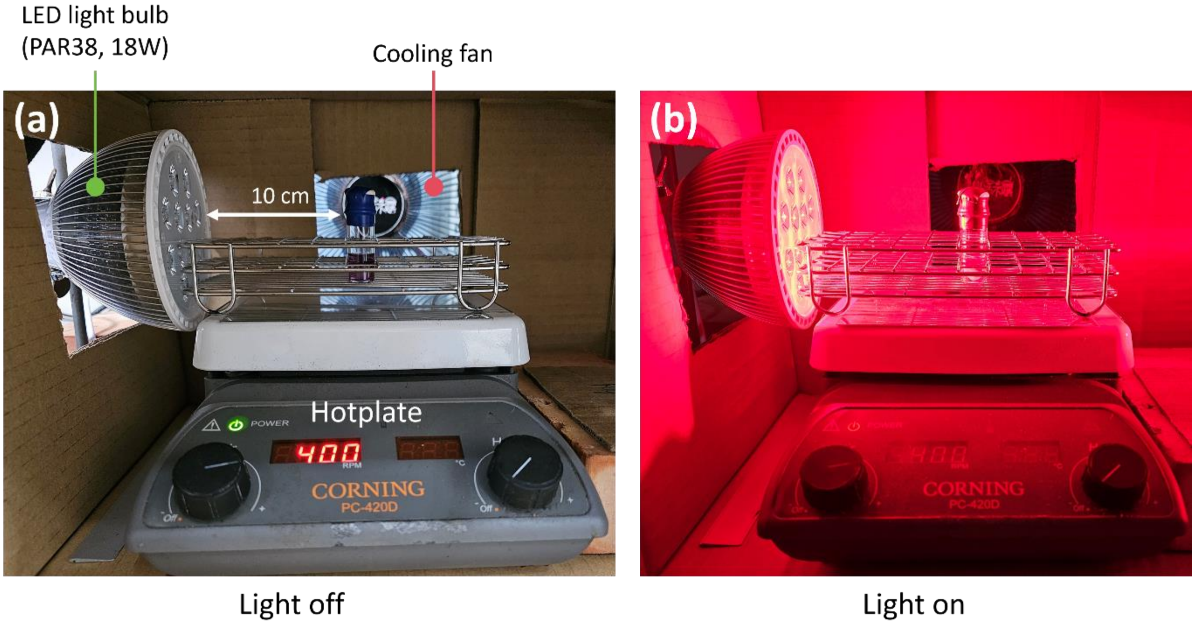

Figure S8. Digital images demonstrating the setup for photopolymerization in the light-off state (a) and the light-on state (b). The scintillation vial was placed 10 cm away from the light source (λ_{max}= 625 nm, I= 35 mW/cm^2), and the temperature of the photopolymerization system was controlled by a cooling fan.

Table S3. Control experiments of PET-RAFT polymerization of ODMMAm under red light irradiation[a]

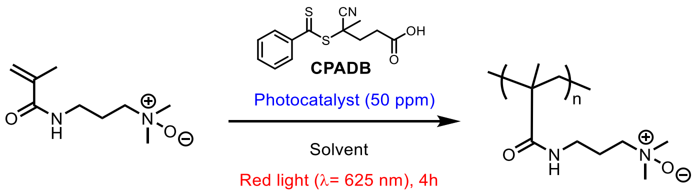

Entry	Catalyst	Solvent	Note	Conv. (%)[b]	$M_{n,th}$ (kDa)[c]	$M_{n,GPC}$ (kDa)[d]	Đ[d]	Dev. (%)[e]
1	ZnTPP	30% H_2O/DMSO	No catalyst	0	---	---	---	---
2			No CTA	0	---	---	---	---
3	$ZnTPS_4$	70% H_2O/DMSO	No catalyst	0	---	---	---	---
4			No CTA	2	---	---	---	---

[a]Polymerization was carried out under deoxygenated conditions with the initial ratio of [M]:[CTA]:[I]= 200:1:0.1, [PC]:[M]= 50 ppm and [M]= 3 M. The polymerization temperature was maintained at 25 °C throughout the experiment; [b]Determined by ^{1}H NMR in D_2O; [c]Theoretical molecular weight was calculated using the following equation: $M_{n,th}$= $[M_o]/[CTA] \times$ conv. $\times M_{w,monomer} + M_{w,RAFT}$, where $[M_o]$, [CTA], conv., $M_{w,monomer}$, and $M_{w,RAFT}$ correspond to initial monomer concentration, initial RAFT agent concentration, monomer conversion determined by ^{1}H NMR, molar mass of monomer, and molar mass of RAFT agent; [d]Molecular weight ($M_{n,GPC}$) and dispersity (Đ) were determined by GPC analysis (TFE as eluent) calibrated using PMMA standards

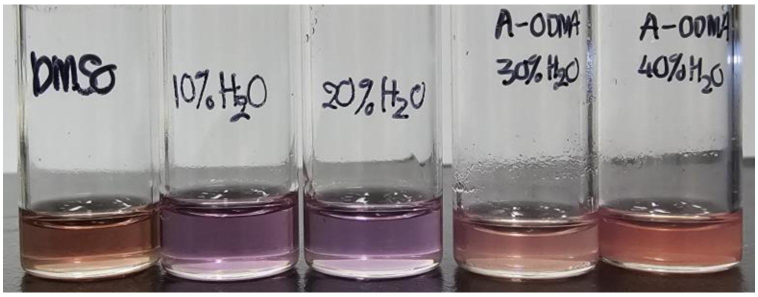

Figure S9. Digital images of the crude reaction mixture after polymerization. The precipitation of the ZnTPP photocatalyst was observed at high water content (> 30 %, v/v), resulting in a slightly opaque solution.

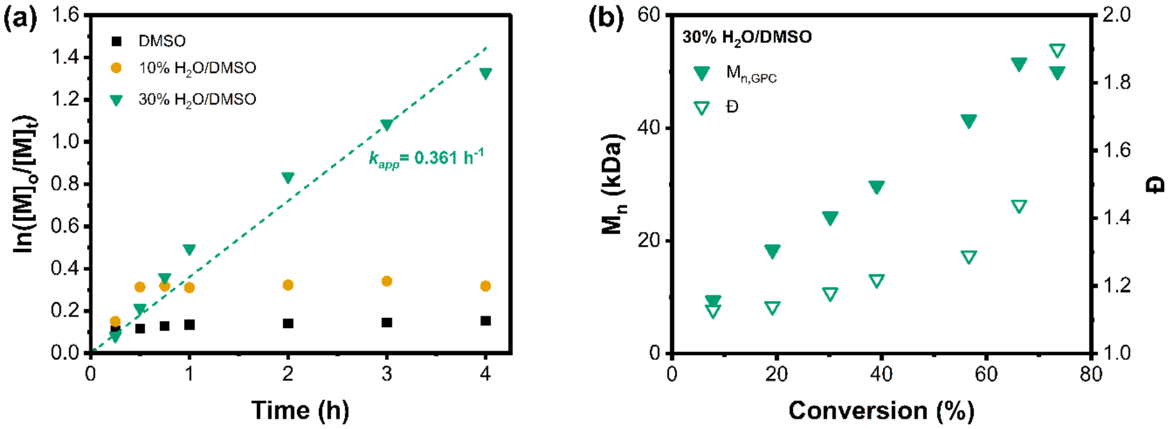

Figure S10. (a) Kinetics study of ZnTPP-mediated PET-RAFT polymerization of ODMMAm in 0, 10, and 30 vol% H_2O/DMSO under red light irradiation. (b) Evolution of Mn and Đ versus monomer conversion for the P(ODMMAm). The polymerization was conducted at the initial ratio of [ODMMAm]:[CPADB]:[ZnTPP]= 200:1:0.01, [ZnTPP]:[ODMMAm]= 50 ppm, and a monomer concentration of 3 M.

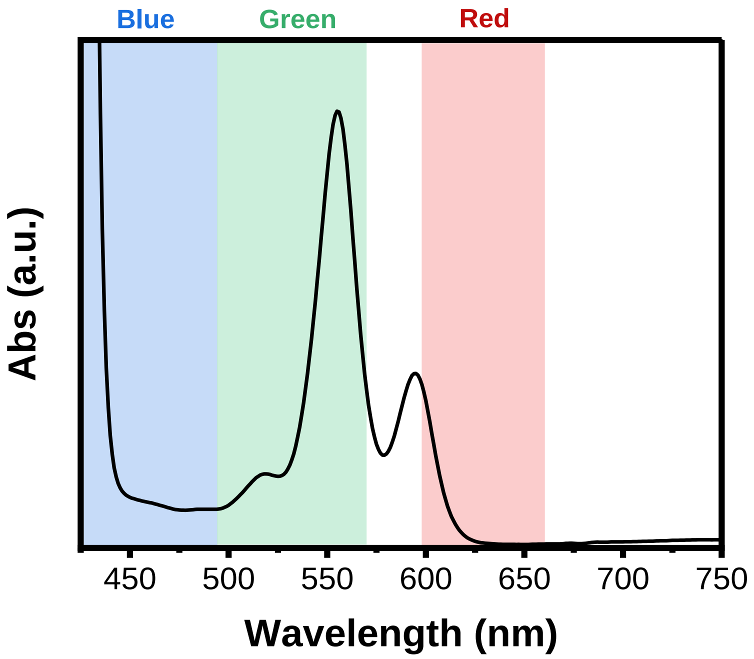

Figure S11. UV-Vis absorption spectrum of ZnTPS4 in H_2O and the emission region of LED light sources used in our studies.

Table S4. Polymerization of ODMMAm using different light wavelengths[a]

Entry	[PC]/[M] (ppm)	Light	λ_{max} (nm)	α (%)[b]	$M_{n,th}$[c] (kDa)	$M_{n,GPC}$[d] (kDa)	Đ[d]	Dev.[e]
1[f]		Red	625	0	---	---	---	---
2	0	Green	520	30	11.5	19.7	1.12	71%
3		Blue	450	53	20.0	30.7	1.22	54%

[a]Reaction conditions: [ODMMAm]:[CPADB]:[ZnTPS4]= 200:1:x, [ODMMAm]= 3 M in 70% H_2O/DMSO, irradiated for 4 hours under different LEDs; [b]Determined by ^{1}H NMR spectroscopy; [c]Theoretical molecular weight was calculated using the following equation: $M_{n,th}$= $[M_o]/[CTA] \times \alpha \times M_{w,monomer} + M_{w,CTA}$, where $[M_o]$, [CTA], α, $M_{w,monomer}$, $M_{w,CTA}$ correspond to initial monomer concentration, initial CTA concentration, monomer conversion determined by ^{1}H NMR, molar mass of monomer, and molar mass of CTA; [d]Molecular weight ($M_{n,GPC}$) and dispersity (Đ) were determined by GPC analysis (TFE as eluent) calibrated using PMMA standards; [e]Deviation (Dev.)= $|M_{n,th} - M_{n,GPC}|/M_{n,th} \times 100\%$; [f]Taken from Table S2, entry 3.

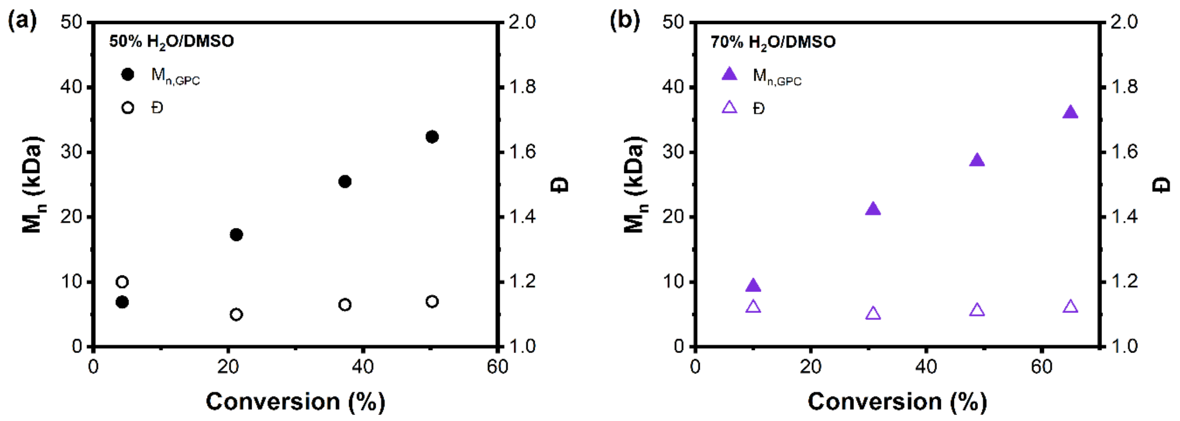

Figure S12. Evolution of Mn and Đ versus monomer conversion for the P(ODMMAm) conducted in (a) 50% H_2O/DMSO and (b) 70% H_2O/DMSO. The polymerization was performed at the initial ratio of [ODMMAm]:[CPADB]:[$ZnTPS_4$]= 200:1:0.01, [$ZnTPS_4$]:[ODMMAm]= 50 ppm, and a monomer concentration of 3 M.

Table S5. Varying the tertiary amine as electron donor in PET-RAFT polymerization of ODMMAm[a]

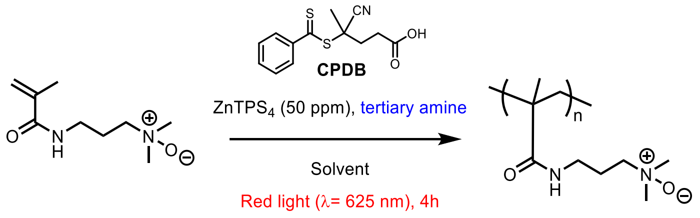

Entry	Note	Conv. (%)[b]	$M_{n,th}$ (kDa)[c]	$M_{n,GPC}$ (kDa)[d]	Đ[d]	Dev. (%)[e]
1	10 eq. of triethylamine (TEA)	98	36.4	55.5	1.44	52
2	10 eq. of triethanolamine (TEOA)	81	30.3	39.9	1.16	32
3	15 eq. of triethanolamine (TEOA)	89	33.5	39.9	1.18	19
4	5 eq. of triethanolamine (TEOA)	85	31.9	39.6	1.14	24

[a]Polymerization was carried out under deoxygenated conditions with the initial ratio of [M]:[CPADB]:[I]= 200:1:0.1, [PC]:[M]= 50 ppm and [M]= 3M in 70% H_2O/DMSO. The polymerization temperature was maintained at 25 °C throughout the experiment and irradiated for 4h under red light; [b]Determined by ^{1}H NMR in D_2O; [c]Theoretical molecular weight was calculated using the following equation: $M_{n,th}$= $[M_o]/[CTA] \times$ conv. $\times M_{w,monomer} + M_{w,RAFT}$, where $[M_o]$, [CTA], conv., $M_{w,monomer}$, and $M_{w,RAFT}$ correspond to initial monomer concentration, initial RAFT agent concentration, monomer conversion determined by ^{1}HNMR, molar mass of monomer, and molar mass of RAFT agent; [d]Molecular weight ($M_{n,GPC}$) and dispersity (Đ) were determined by GPC analysis (TFE as eluent) calibrated using PMMA standards

Table S6. Control experiments to reveal the role of TEOA in PET-RAFT polymerization[a]

Entry	Monomer	Deoxygenation	[ZnTPS4] (mol% to monomer)	[TEOA] (mol% to monomer)	Time (h)	α [b] (%)	$M_{n,th}$ [c] (kDa)	$M_{n,GPC}$ [d] (kDa)	Đ[d]
1[e]		Yes	0	2.5	4	0	---	---	---
2	ODMMAm	Yes	0.005	2.5	4	0	---	---	---
3		No	0.005	2.5	4	0	---	---	---
4[f]		Yes	0	2.5	1	0	---	---	---
5	ODMAm	Yes	0.005	2.5	1	0	---	626.3[g]	---
6		No	0.005	2.5	1	0	---	667.7[g]	---

[a]Reaction conditions: [M]:[ZnTPS4]:[TEOA]= 200:0.01:5, [M]= 3.0 M in 70% H_2O/DMSO, irradiated with red light (λ_{max}= 625 nm, I= 35 mW/cm^2).

[b]Monomer conversion was determined from the ^{1}H NMR spectroscopy

[c]Theoretical molecular weight was determined from the monomer conversion

[d]Number-average molecular weight ($M_{n,GPC}$) and dispersity (Đ) were determined by GPC with TFE + 20 mM CF_3COONa as eluent at 0.4 mL/min by using PMMA as standard

[e]CPADB was used as RAFT agent

[f]CBPA was used as RAFT agent

[g]Peak molecular weight (M_p) was reported because a portion of peak is beyond our column range

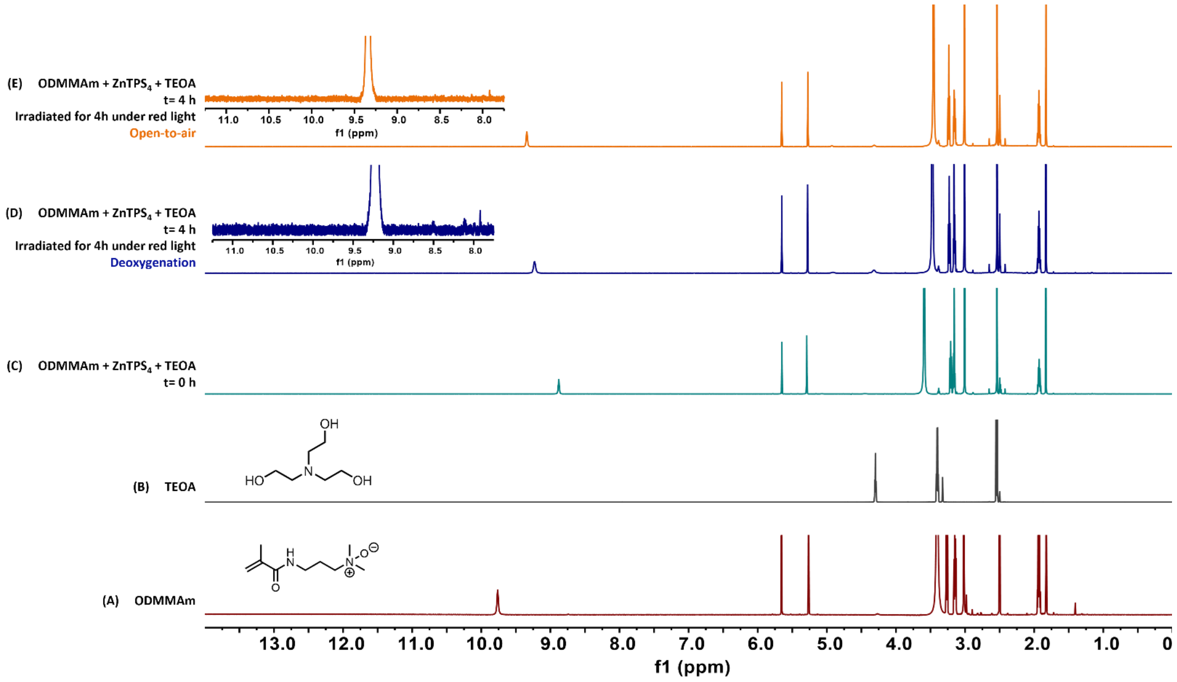

Figure S13. Stacked ^{1}H NMR (600 MHz, DMSO-d_6) of ODMMAm monomer (A), TEOA (B), a polymerization mixture in the absence of RAFT agent before irradiation (C), a polymerization mixture irradiated for 4h with red light in deoxygenation conditions (D), and a polymerization mixture irradiated for 4h with red light in open-to-air conditions (E).

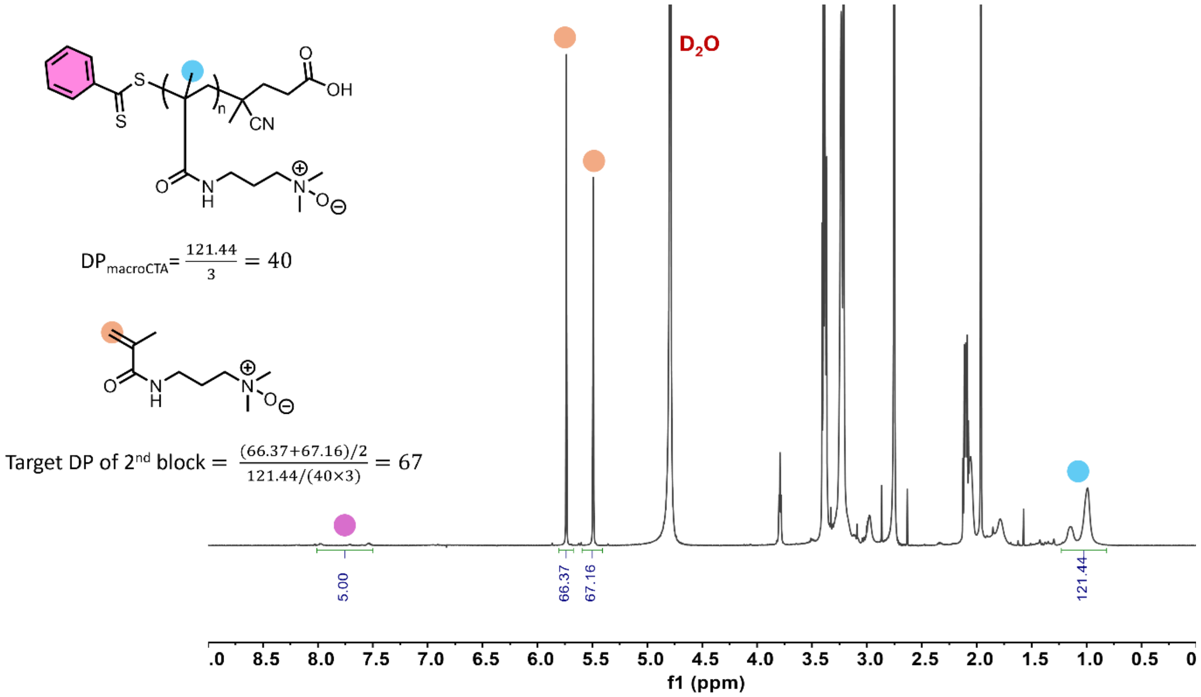

Figure S14. ^{1}H NMR spectrum (600 MHz, D_2O) of P(ODMMAm)$_{40}$-*b*-P(ODMMAm)$_{67}$ before irradiation (t= 0 h)

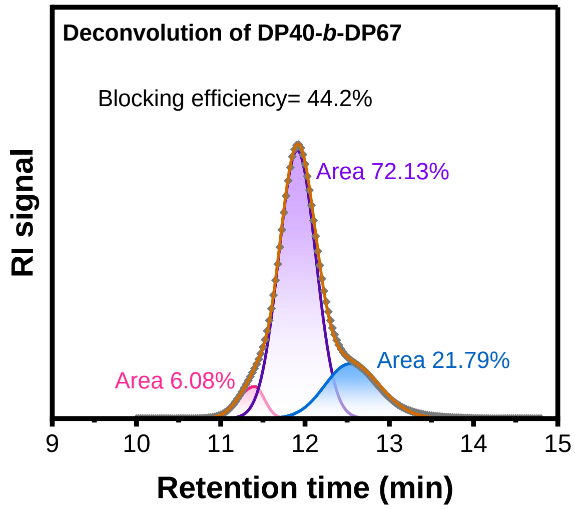

Figure S15. GPC deconvolution data of chain-extension studies for P(ODMMAm)$_{40}$-*b*-P(ODMMAm)$_{67}$. The blocking efficiency was calculated as shown in the equation S1.

$$B_{eff} = \frac{\frac{A_{copolymer}}{DP_{final}}}{\frac{A_{copolymer}}{DP_{final}} + \frac{A_{final,macro-CTA}}{DP_{macro-CTA}}} = \frac{\frac{53.9326}{167}}{\frac{53.9326}{167} + \frac{16.2931}{40}} = 0.442$$

Equation S1. Calculation of blocking efficiency of P(ODMMAm)40-b-P(ODMMAm)67 block copolymers, based on the integration of GPC deconvolution peaks in Figure S12

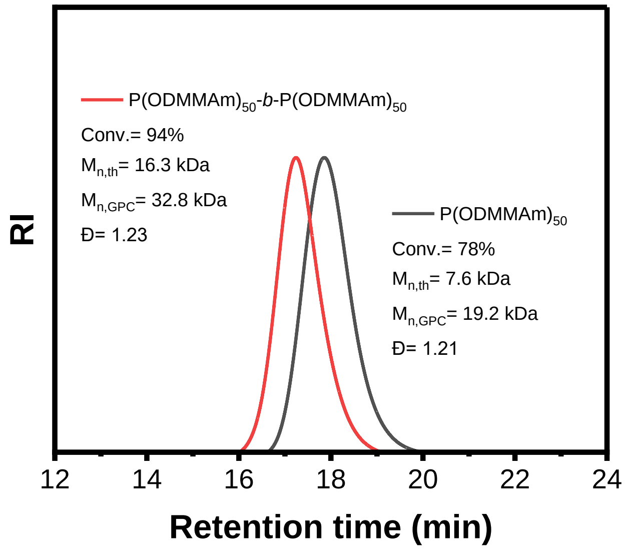

Figure S16. GPC chromatogram of P(ODMMAm)$_{50}$-*b*-P(ODMMAm)$_{50}$ synthesized by *in situ* block copolymerization. The feed ratio for the first block was [ODMMAm]:[CPDT]:[ZnTPS$_4$]:[TEOA]= 50:1:0.0025:5, [M]= 3.0 M in 70% H_2O/DMSO. The GPC flow rate was 0.4 mL/min.

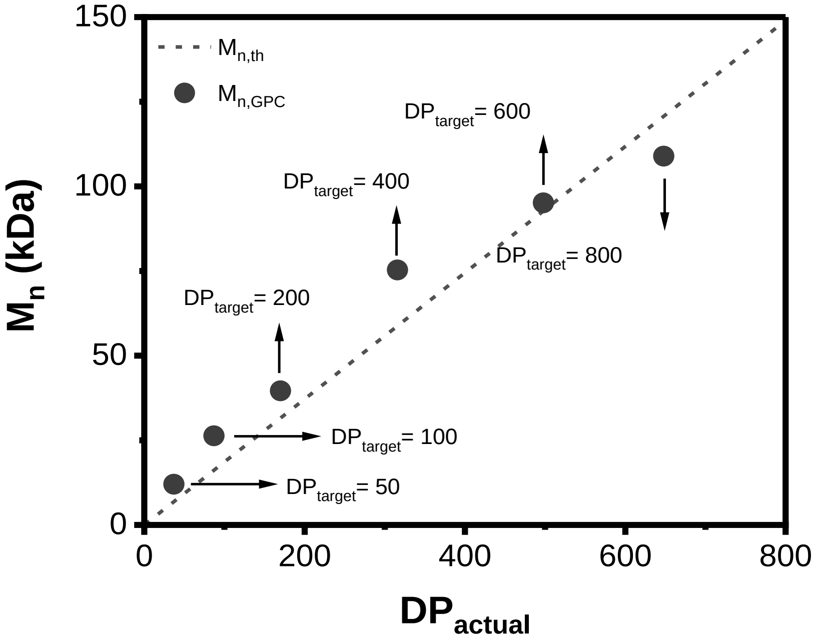

Figure S17. Evolution of P(ODMMAm) molecular weight at different target DPs under red-light induced PET-RAFT polymerization in 70% H_2O/DMSO.

Table S7. Varying the target DPs on PET-RAFT polymerization of ODMMAm under red light irradiation[a]

Entry	Target DPs	Conv. (%)[b]	$M_{n,th}$ (kDa)[c]	$M_{n,GPC}$ (kDa)[d]	Đ[d]	Dev. (%)[e]
1	25	74	3.7	6.4	1.14	73
2	50	79	7.6	12.0	1.09	58
3	100	87	16.5	26.3	1.13	60
4	200	83	31.2	39.6	1.14	27
5	400	79	59.1	75.3	1.18	27
6	600	83	93.0	95.1	1.22	2
7	800	81	121.0	108.9	1.25	10

[a]Polymerization was carried out under deoxygenated conditions with the initial ratio of [M]:[CTA]:[I]:[TEOA]= x:1:0.01:5, where x is a target degree of polymerization (DP), [PC]:[M]= 50 ppm and [M]= 3M in 70% H_2O/DMSO. The polymerization temperature was maintained at 25°C throughout the experiment and irradiated for 4h under red light; [b]Determined by ^{1}H NMR in D_2O; [c]Theoretical molecular weight was calculated using the following equation: $M_{n,th}$= $[M_o]/[CTA] \times conv. \times M_{w,monomer} + M_{w,RAFT}$, where $[M_o]$, [CTA], conv., $M_{w,monomer}$, and $M_{w,RAFT}$ correspond to initial monomer concentration, initial RAFT agent concentration, monomer conversion determined by ^{1}H NMR, molar mass of monomer, and molar mass of RAFT agent; [d]Molecular weight ($M_{n,GPC}$) and dispersity (Đ) were determined by GPC analysis (TFE as eluent) calibrated using PMMA standards

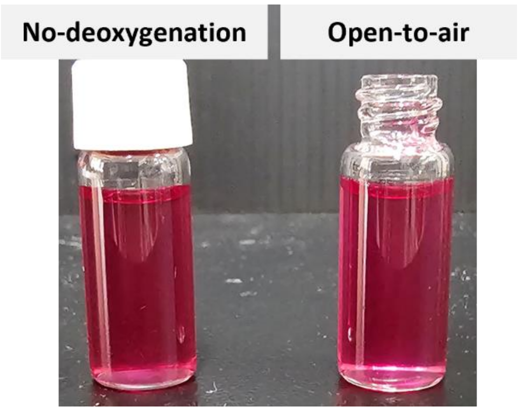

Figure S18. Digital image of the polymerization set-up for oxygen-tolerant evaluation: capped vial without deoxygenation (left) and opened vial.

Table S8. Evaluate the oxygen-tolerance of PET-RAFT polymerization of ODMMAm under red light[a]

Entry	Note	Conv. (%)[b]	$M_{n,th}$ (kDa)[c]	$M_{n,GPC}$ (kDa)[d]	Đ[d]	Dev. (%)[e]
1	Deoxygenation	85	31.9	39.6	1.14	24
2	Capped vial – No deoxygenation	77	28.9	31.5	1.14	9
3	Open-to-air	77	28.9	29.8	1.15	3

[a]Polymerization was carried out under deoxygenated conditions with the initial ratio of [M]:[CTA]:[I]= 200:1:0.1, [PC]:[M]= 50 ppm and [M]= 3M in 70% H_2O/DMSO. The polymerization temperature was maintained at 25°C throughout the experiment and irradiated for 4h under red light; [b]Determined by ^{1}H NMR in D_2O; [c]Theoretical molecular weight was calculated using the following equation: $M_{n,th}$= $[M_o]/[CTA] \times conv. \times M_{w,monomer} + M_{w,RAFT}$, where $[M_o]$, [CTA], conv., $M_{w,monomer}$, and $M_{w,RAFT}$ correspond to initial monomer concentration, initial RAFT agent concentration, monomer conversion determined by ^{1}HNMR, molar mass of monomer, and molar mass of RAFT agent; [d]Molecular weight ($M_{n,GPC}$) and dispersity (Đ) were determined by GPC analysis (TFE as eluent) calibrated using PMMA standards

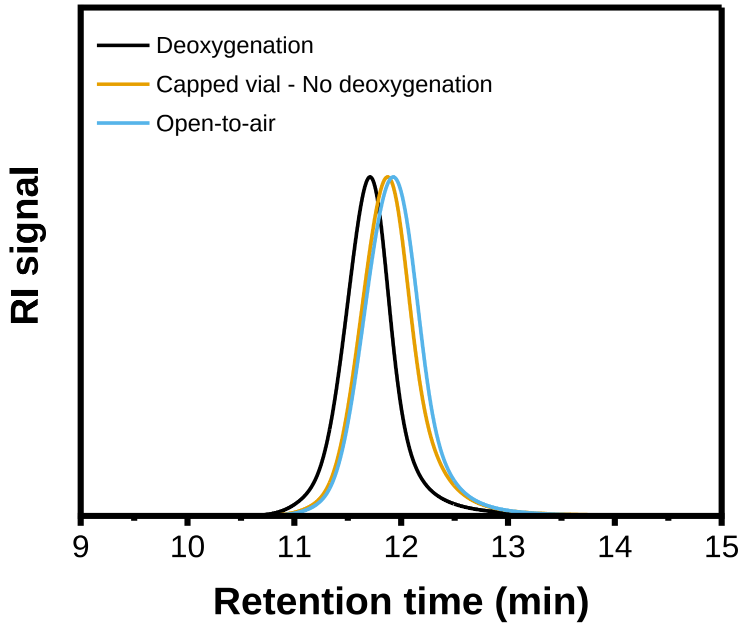

Figure S19. GPC traces of P(ODMMAm) were synthesized under deoxygenation, capped vial – no deoxygenation and open-to-air environment red-light irradiation in corresponding to Table S6. Polymerization was performed under the following conditions: [ODMMAm]:[CPADB]:[ZnTPS4]:[TEOA]= 200:1:0.01:5, [ZnTPS4]:[ODMMAm]= 50 ppm, [ODMMAm]= 3 M in 70% H_2O/DMSO. The mixture was irradiated under red light for 4h.

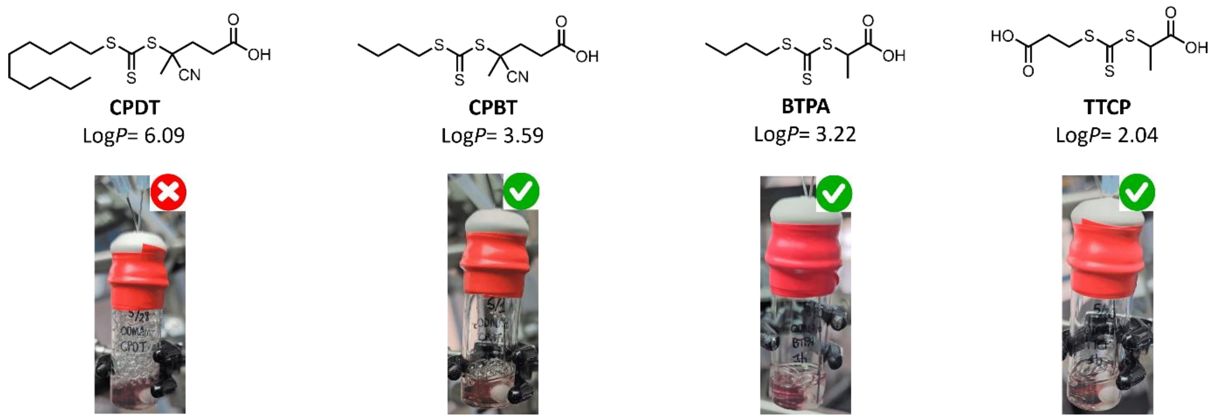

Figure S20. Digital images of the degassing process by bubbling N_2 in the presence of different trithiocarbonate CTAs. The amphiphilic CPDT forms a stable foam during the degassing process, which was not observed in other hydrophilic trithiocarbonates.

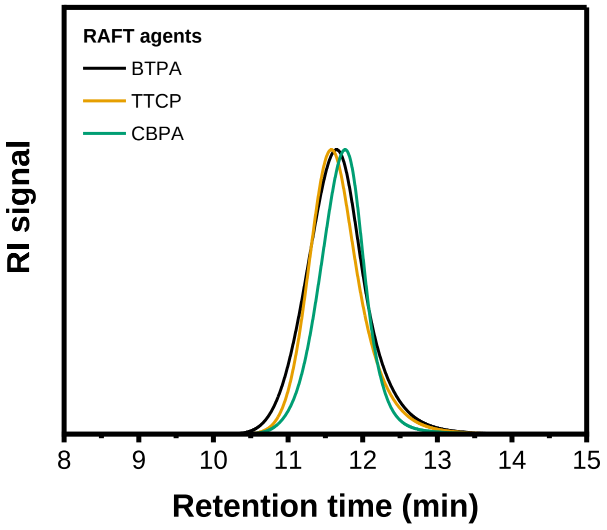

Figure S21. GPC traces of P(ODMAm) synthesized by using different RAFT agents under red-light induced PET-RAFT polymerization in 70% H_2O/DMSO, corresponding to Table 3.

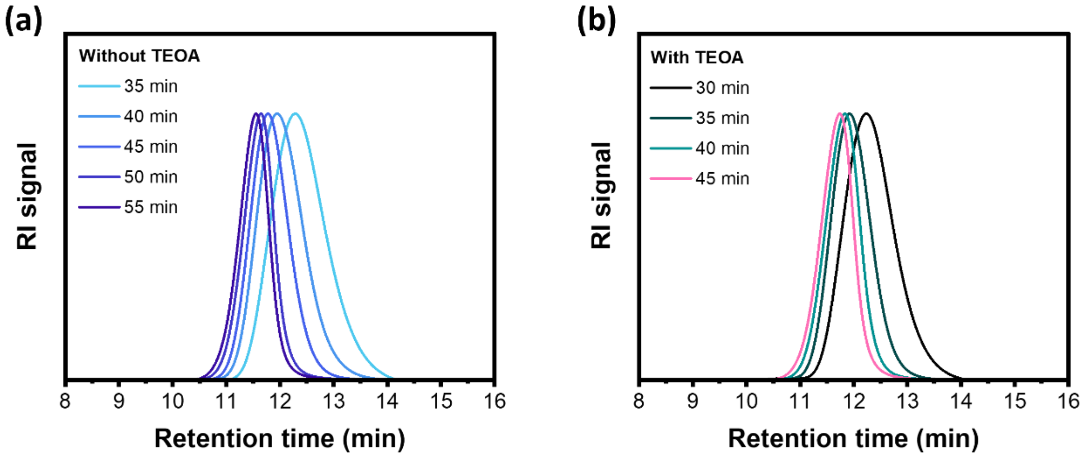

Figure S22. GPC traces of P(ODMAm) synthesized in the presence or absence of triethanolamine (TEOA).

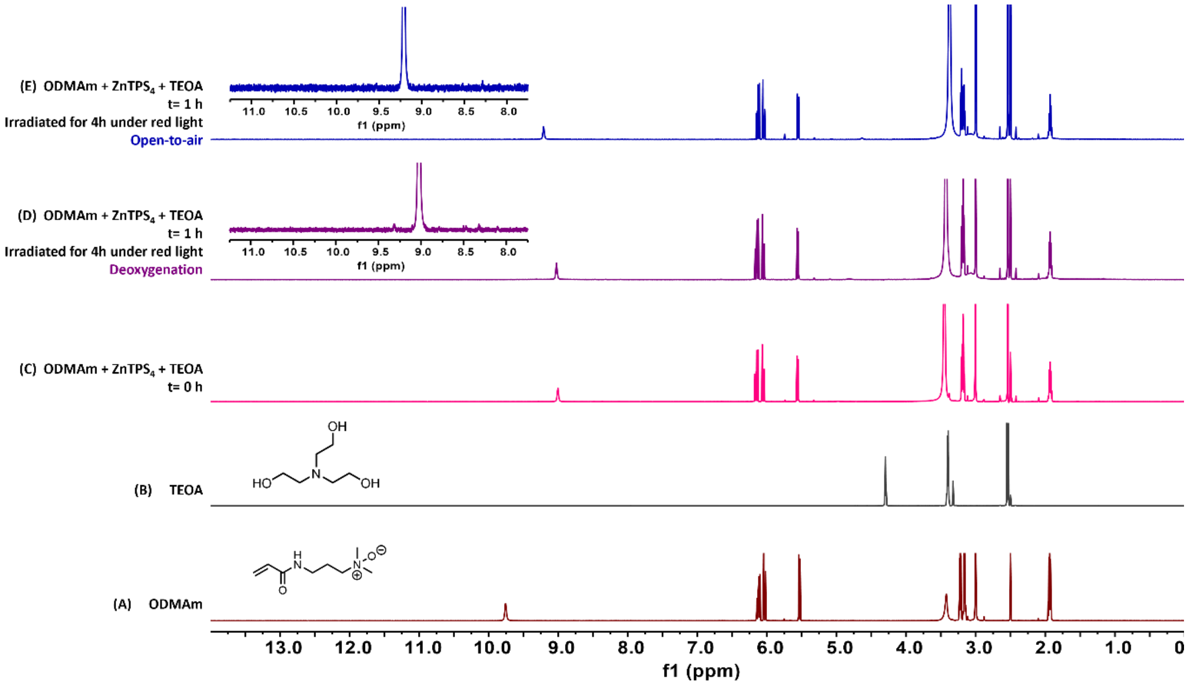

Figure S23. Stacked ^{1}H NMR (600 MHz, DMSO-d_6) of ODMAm monomer (A), TEOA (B), a polymerization mixture in the absence of RAFT agent before irradiation (C), a polymerization mixture irradiated for 4h with red light in deoxygenation conditions (D), and a polymerization mixture irradiated for 4h with red light in open-to-air conditions (E).

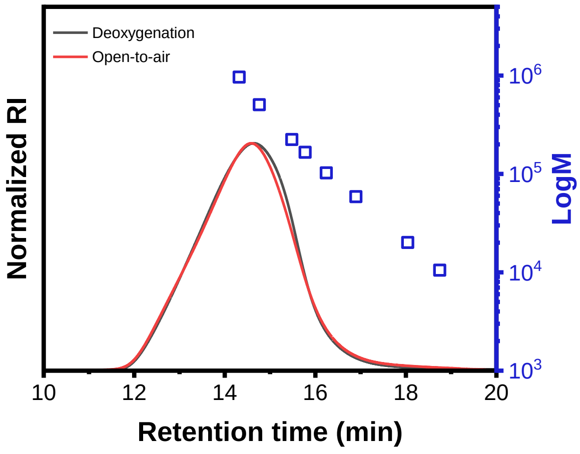

Figure S24. GPC chromatogram of P(ODMAm) obtained from control experiments in the absence of RAFT agent, as shown in entry 5 and 6 of Table S6. The GPC flow rate was 0.4 mL/min.

Table S9. Varying the target DPs on PET-RAFT polymerization of ODMAm under red light irradiation[a]

Entry	Target DPs	Conv. (%)[b]	$M_{n,th}$ (kDa)[c]	$M_{n,GPC}$ (kDa)[d]	Đ[d]	Dev. (%)[e]
1	50	84	7.5	13.7	1.12	83
2	100	88	15.4	23.4	1.12	52
3	200	96	33.5	38.4	1.19	14
4	400	94	65.0	71.0	1.14	9
5	600	86	89.2	108.7	1.17	22
6	800	86	118.8	126.6	1.24	7

[a]Polymerization was carried out under deoxygenated conditions with the initial ratio of [M]:[CBPA]:[PC]:[TEOA]= x:1:0.01:5, where x is a target degree of polymerization (DP), [PC]:[M]= 50 ppm and [M]= 3M in 70% H_2O/DMSO. The polymerization temperature was maintained at 25°C throughout the experiment and irradiated for 1h under red light; [b]Determined by ^{1}H NMR in D_2O; [c]Theoretical molecular weight was calculated using the following equation: $M_{n,th}$= $[M_o]/[CTA]$ × conv. × $M_{w,monomer}$ + $M_{w,RAFT}$, where $[M_o]$, [CTA], conv., $M_{w,monomer}$, and $M_{w,RAFT}$ correspond to initial monomer concentration, initial RAFT agent concentration, monomer conversion determined by ^{1}H NMR, molar mass of monomer, and molar mass of RAFT agent; [d]Molecular weight ($M_{n,GPC}$) and dispersity (Đ) were determined by GPC analysis (TFE as eluent) calibrated using PMMA standards

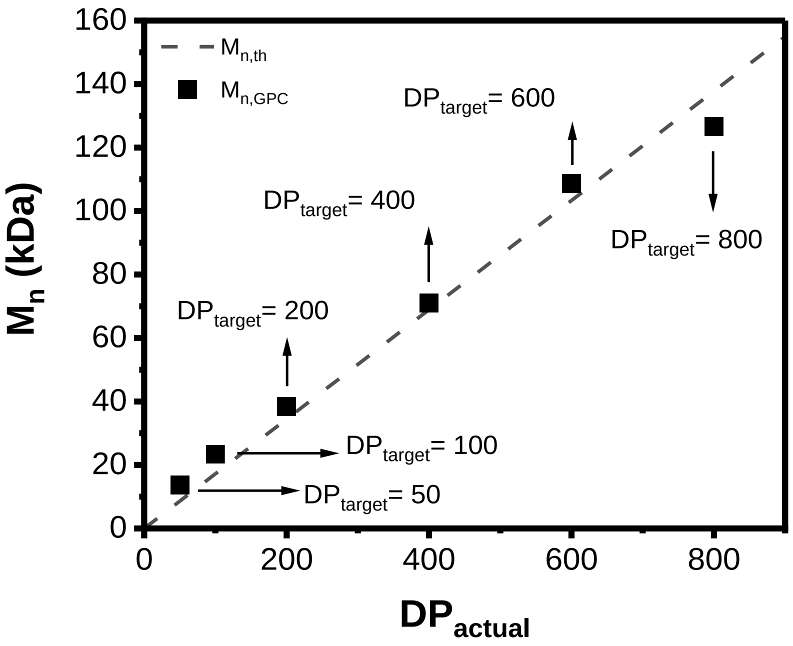

Figure S25. Evolution of P(ODMAm) molecular weight at different target DPs under red-light induced PET-RAFT polymerization in 70% H_2O/DMSO.

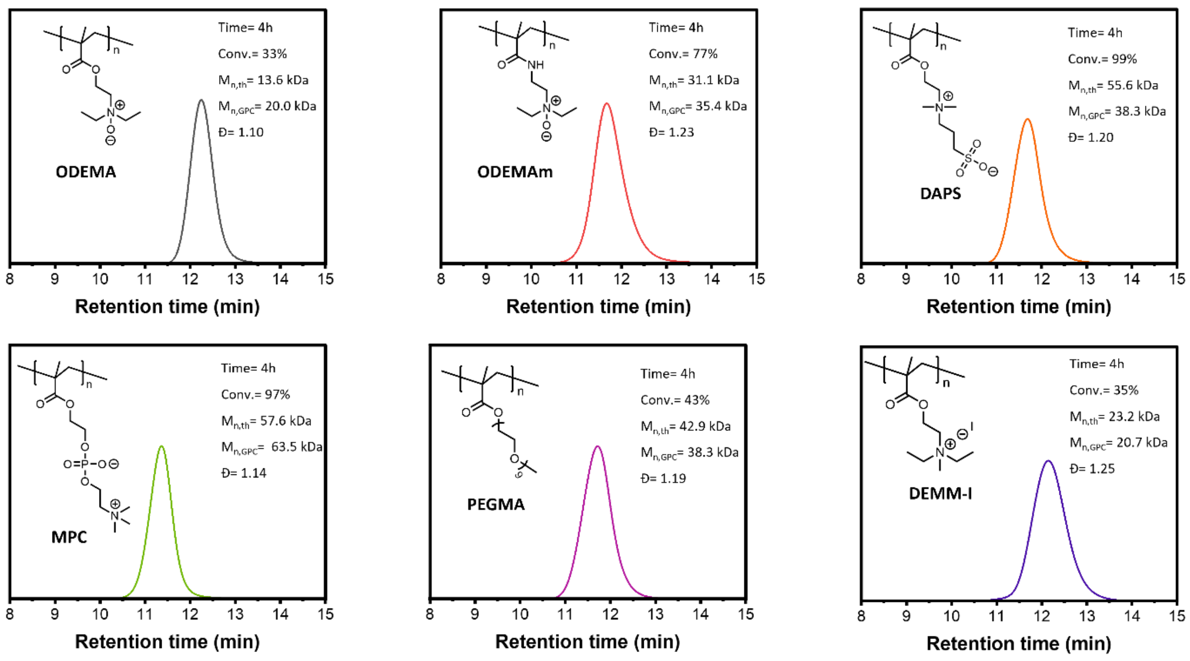

Figure S26. GPC traces of polymers corresponding to Table 5: Vinyl monomer scope of $ZnTPS_4$-mediated PET-RAFT polymerization under red light irradiation

Table S10. Scalability of PET-RAFT polymerization of ODMAm with red light irradiation under open-to-air conditions[a]

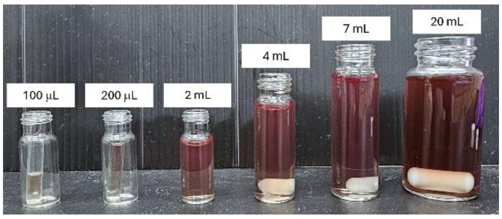

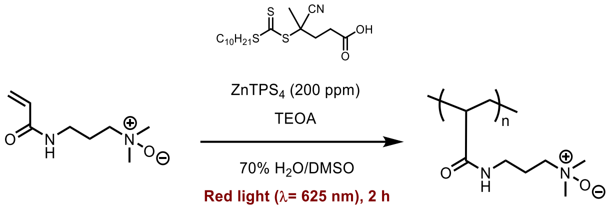

Entry	Volume (μL)	Stirring (rpm)	Conv. (%)[b]	$M_{n,th}$ (kDa)[c]	$M_{n,GPC}$ (kDa)[d]	Đ[d]
1	100	0	92	32.0	76.6	1.20
2	200	0	84	29.3	69.4	1.18
3	2,000	0	92	32.0	62.8	1.18
4	4,000	500	66	23.1	48.2	1.23
5	7,000	500	76	26.6	48.6	1.22
6[e]	20,000	500	0	---	---	---
7[e,f]	20,000	500	97	33.8	73.5	1.26

[a]Polymerization was carried out under deoxygenated conditions with the initial ratio of [M]:[CPDT]:[PC]:[TEOA]= 200:1:0.04:5, [PC]:[M]= 200 ppm and [M]= 0.5 M in 70% H_2O/DMSO. The polymerization temperature was maintained at 25 °C throughout the experiment and irradiated for 1h under red light; [b]Determined by ^1H NMR in D_2O; [c]Theoretical molecular weight was calculated using the following equation: $M_{n,th}$= $[M_o]/[CTA] \times conv. \times M_{w,monomer} + M_{w,RAFT}$, where $[M_o]$, [CTA], conv., $M_{w,monomer}$, and $M_{w,RAFT}$ correspond to initial monomer concentration, initial RAFT agent concentration, monomer conversion determined by ^1H NMR, molar mass of monomer, and molar mass of RAFT agent; [d]Molecular weight ($M_{n,GPC}$) and dispersity (Đ) were determined by GPC analysis (TFE as eluent) calibrated using PMMA standards at the flow rate of 0.4 mL/min; [e]Irradiated for 2 hours under red light; [f]Polymerization was performed in a closed vial without external deoxygenation methods.

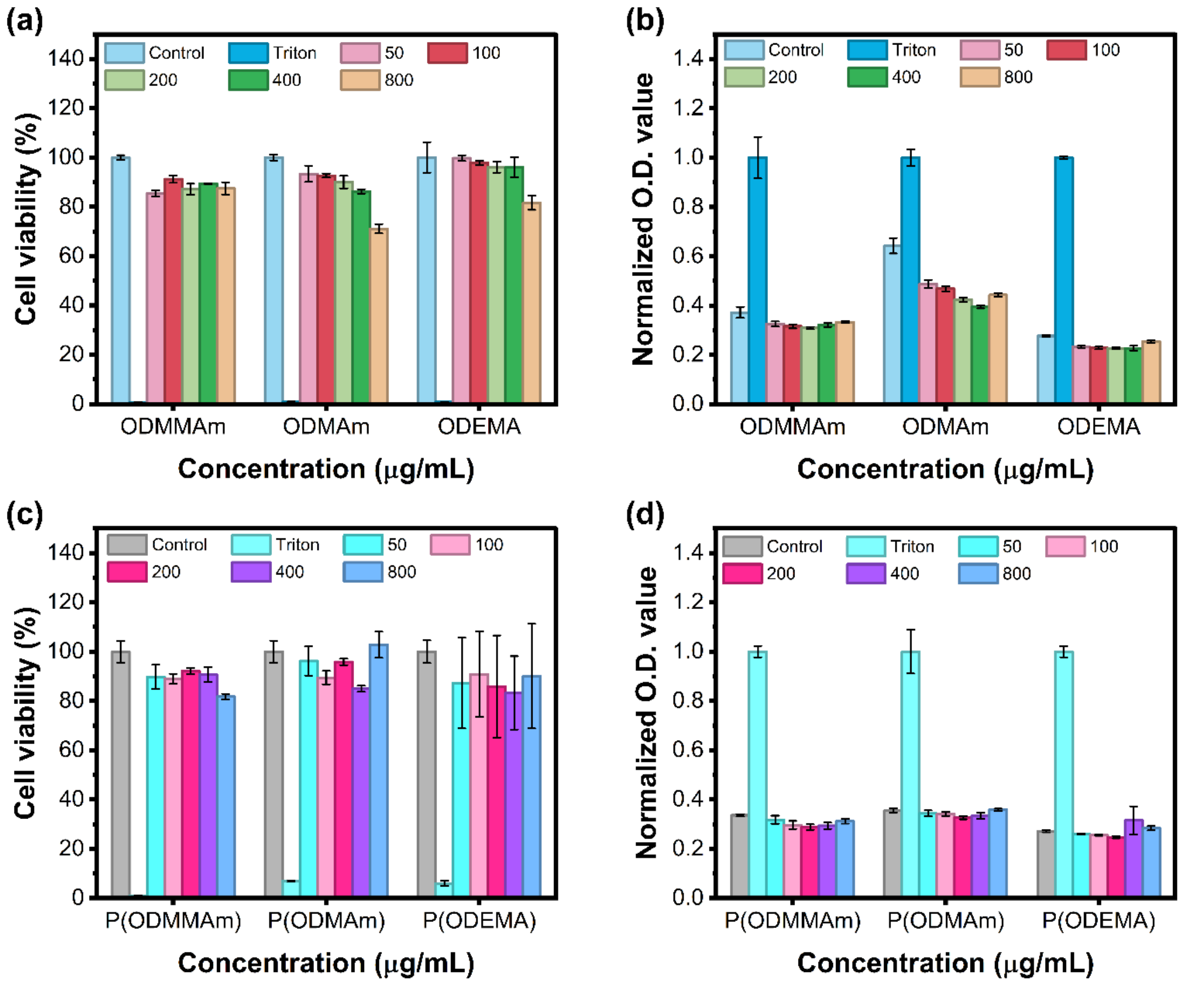

Figure S27. (a) MTT assay for cell viability of L929 fibroblasts treated with ODMMAm, ODMAm, and ODEMA monomers; (b) LDH assay for cell apoptosis of L929 fibroblasts treated with ODMMAm, ODMAm, and ODEMA monomers; (c) MTT assay for cell viability of L929 fibroblasts treated with the synthesized P(ODMMAm), P(ODMAm), and P(ODEMA) polymers; (d) LDH assay for cell apoptosis of L929 fibroblasts treated with the synthesized P(ODMMAm), P(ODMAm), and P(ODEMA) polymers.

4. Characterization details of the synthesized compounds

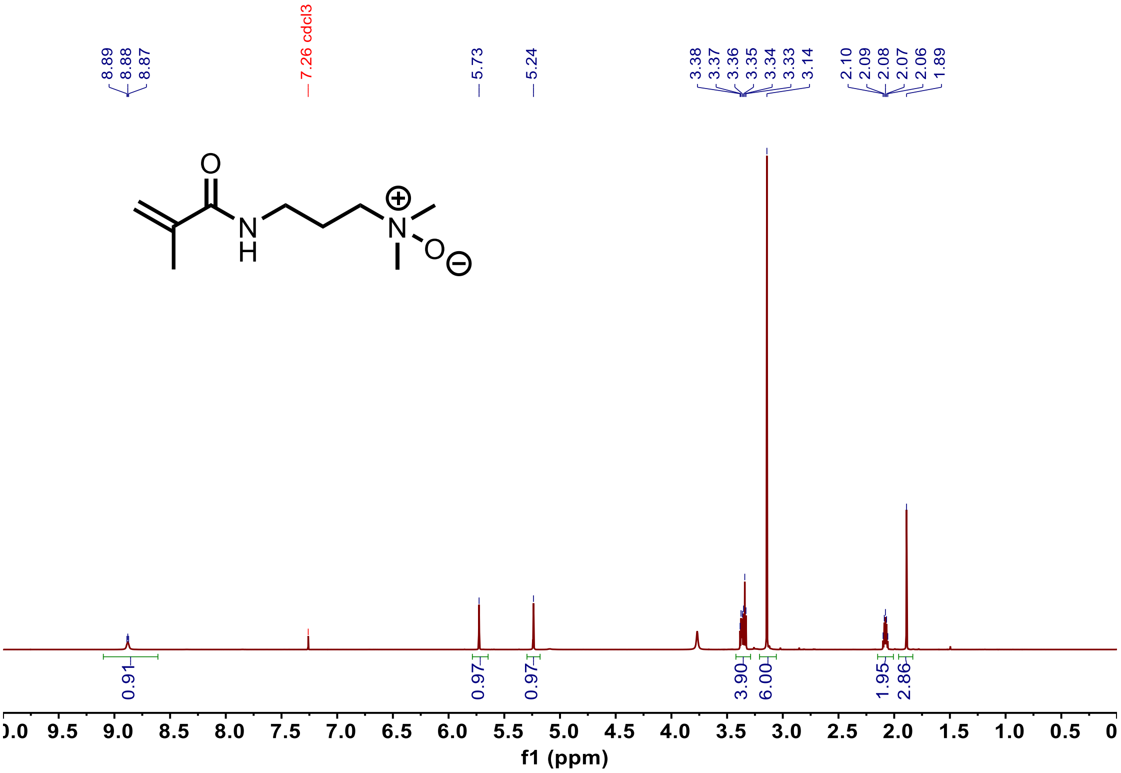

Figure S28. ^{1}H NMR (600 MHz, $CDCl_3$) of *N*-oxide-3-(*N*,*N*-dimethylamino)propyl methacrylamide

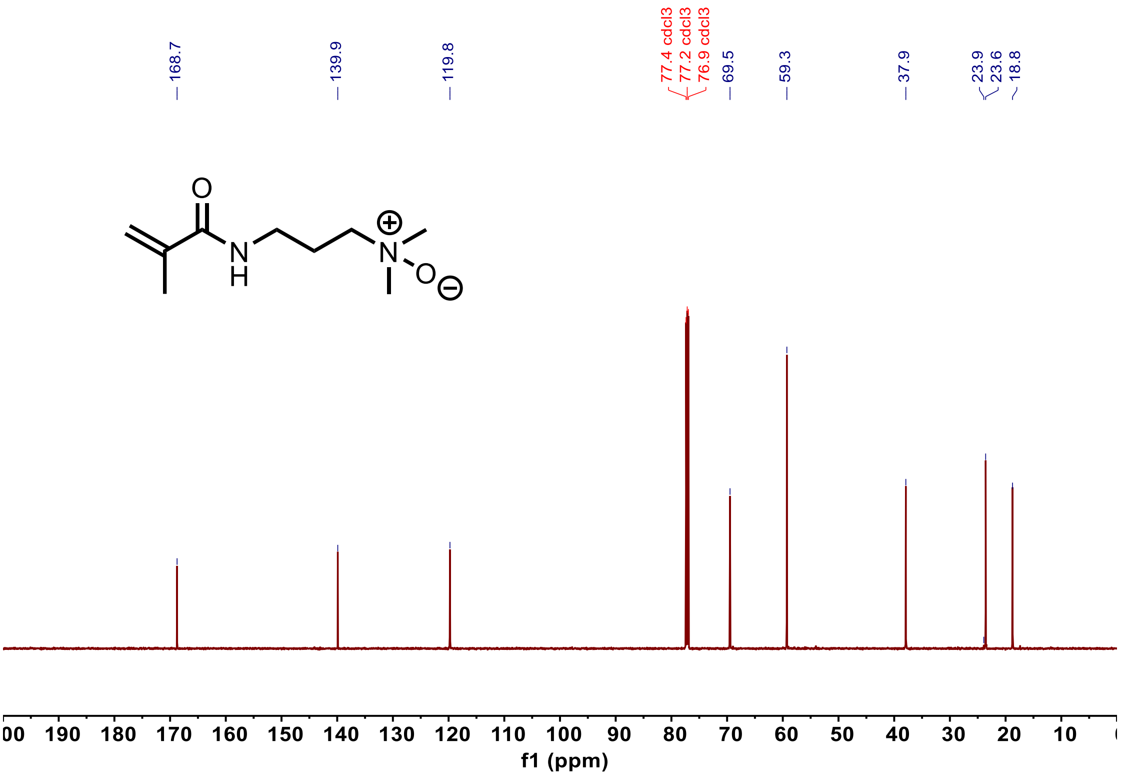

Figure S29. ^{13}C NMR (150 MHz, $CDCl_3$) of *N*-oxide-3-(*N*,*N*-dimethylamino)propyl methacrylamide

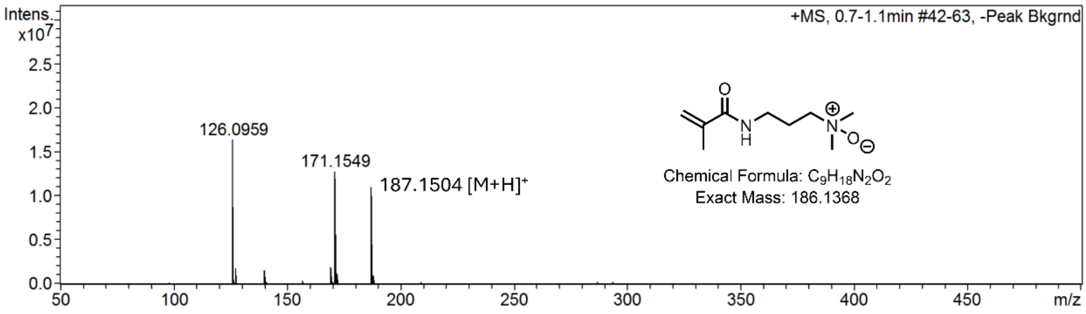

Figure S30. HR-ESI MS of *N*-oxide-3-(*N*,*N*-dimethylamino)propyl methacrylamide

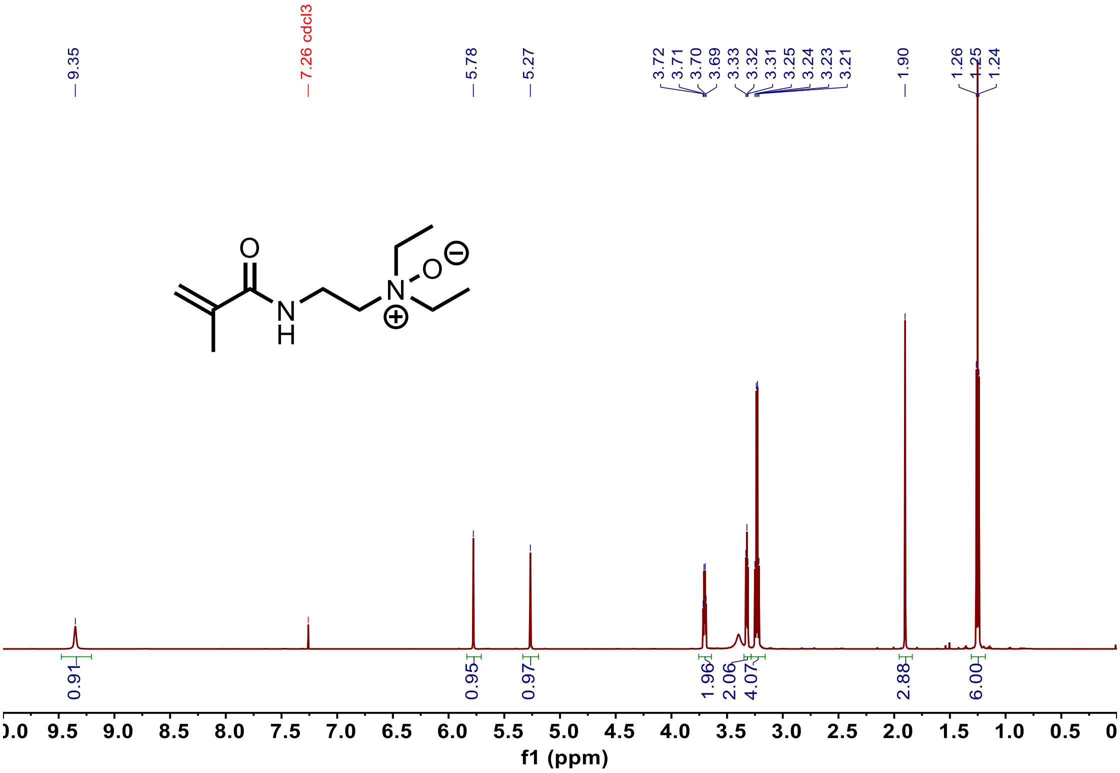

Figure S31. ^{1}H NMR (600 MHz, $CDCl_3$) of *N*-oxide-2-(*N*,*N*-diethylamino)ethyl methacrylamide

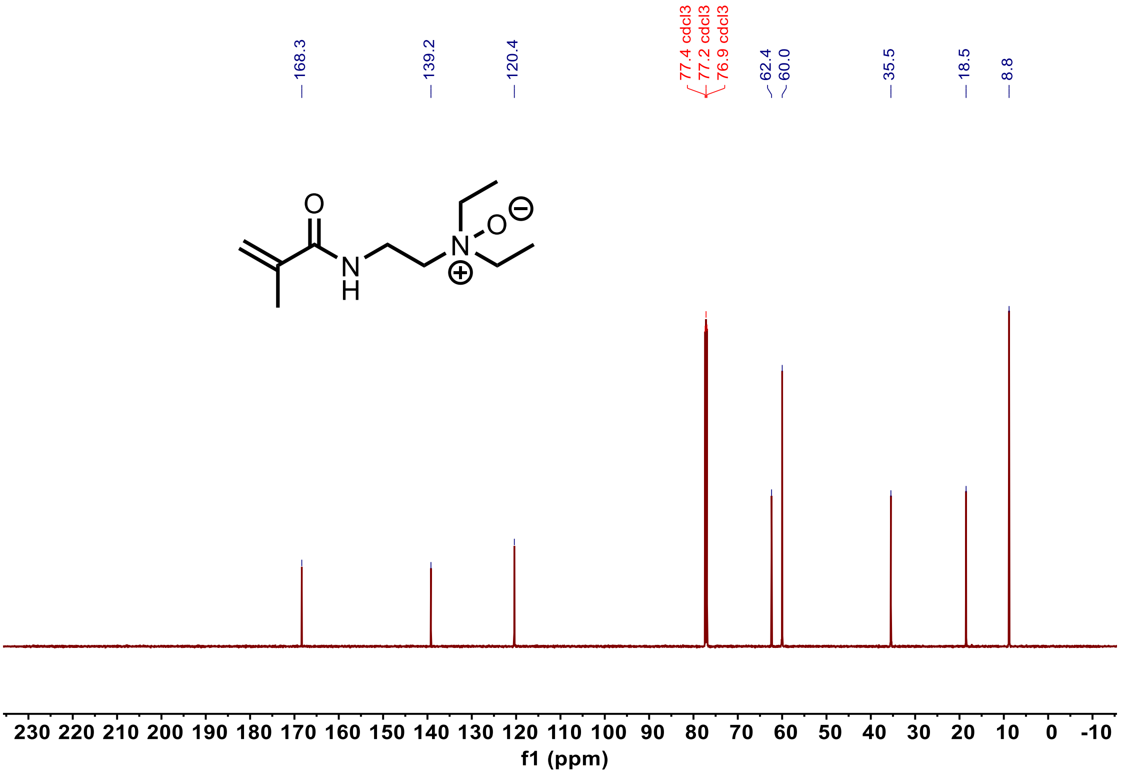

Figure S32. ^{13}C NMR (150 MHz, $CDCl_3$) of *N*-oxide-2-(*N*,*N*-diethylamino)ethyl methacrylamide

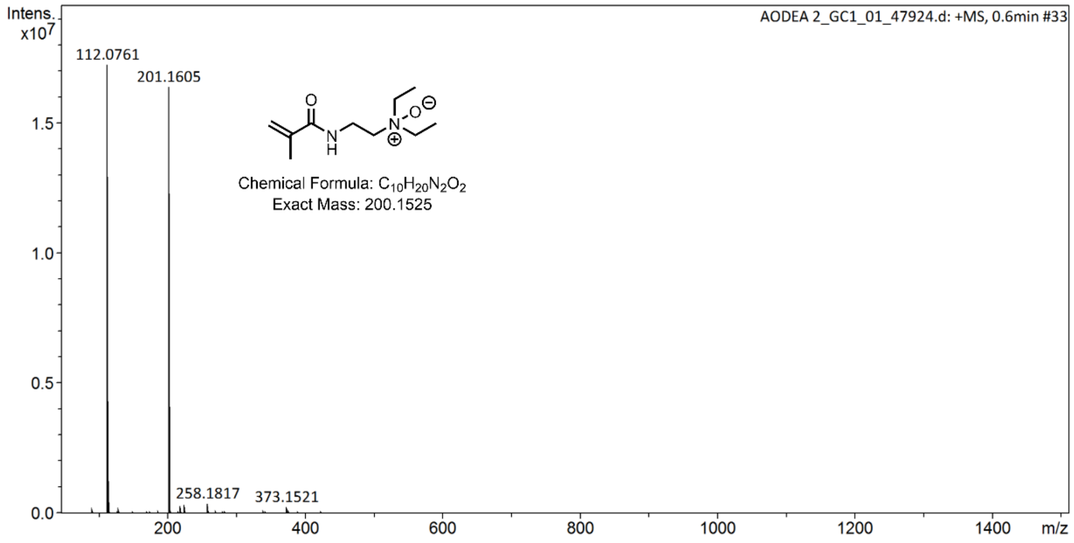

Figure S33. HR-ESI MS of *N*-oxide-2-(*N*,*N*-diethylamino)ethyl methacrylamide

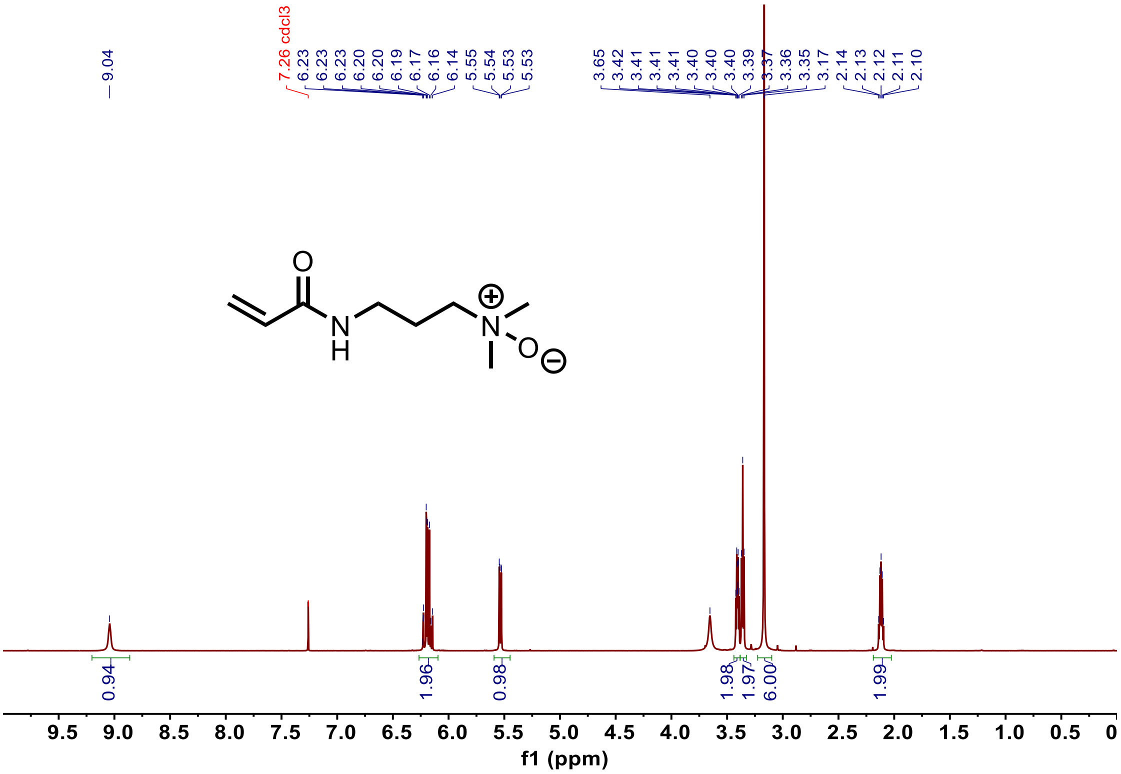

Figure S34. ^{1}H NMR (600 MHz, $CDCl_3$) of *N*-oxide-3-(*N*,*N*-dimethylamino)propyl acrylamide

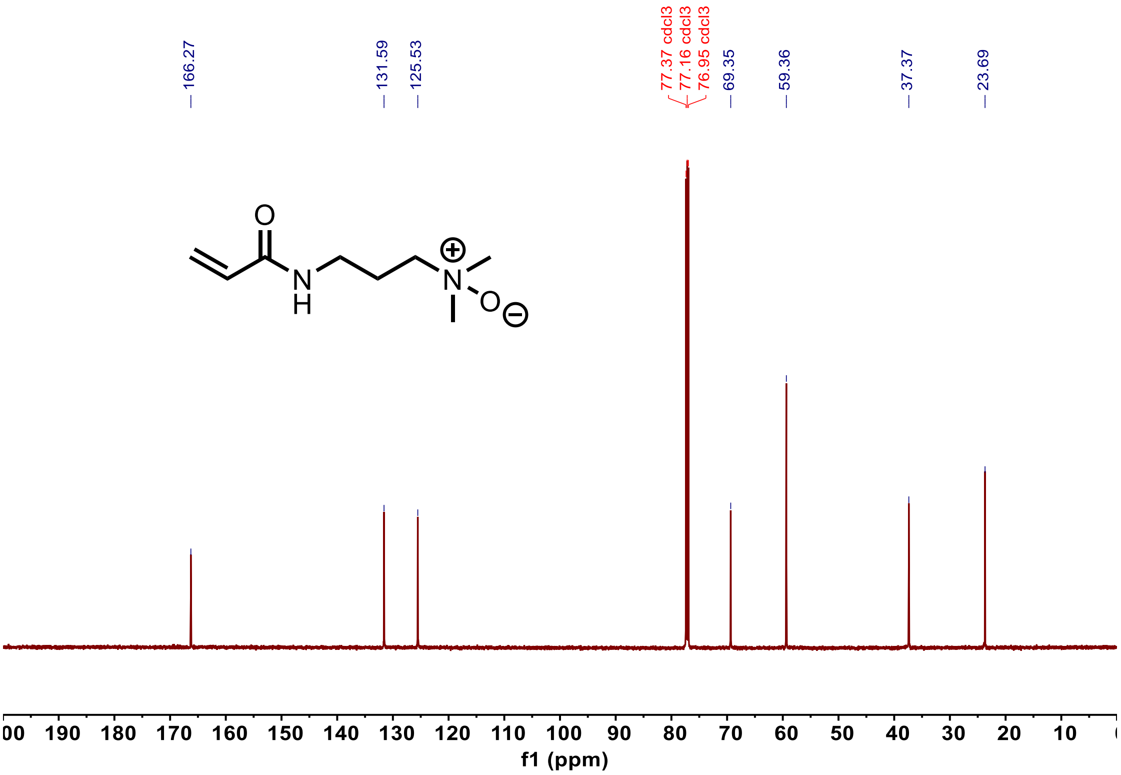

Figure S35. ^{13}C NMR (150 MHz, $CDCl_3$) of *N*-oxide-3-(*N*,*N*-dimethylamino)propyl acrylamide

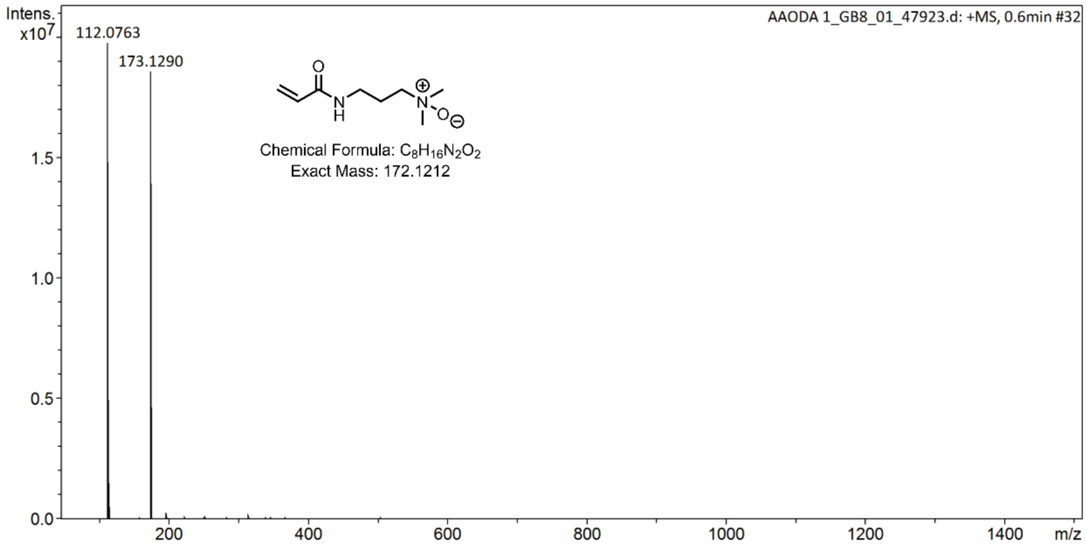

Figure S36. HR-ESI MS of *N*-oxide-2-(*N*,*N*-diethylamino)ethyl methacrylamide

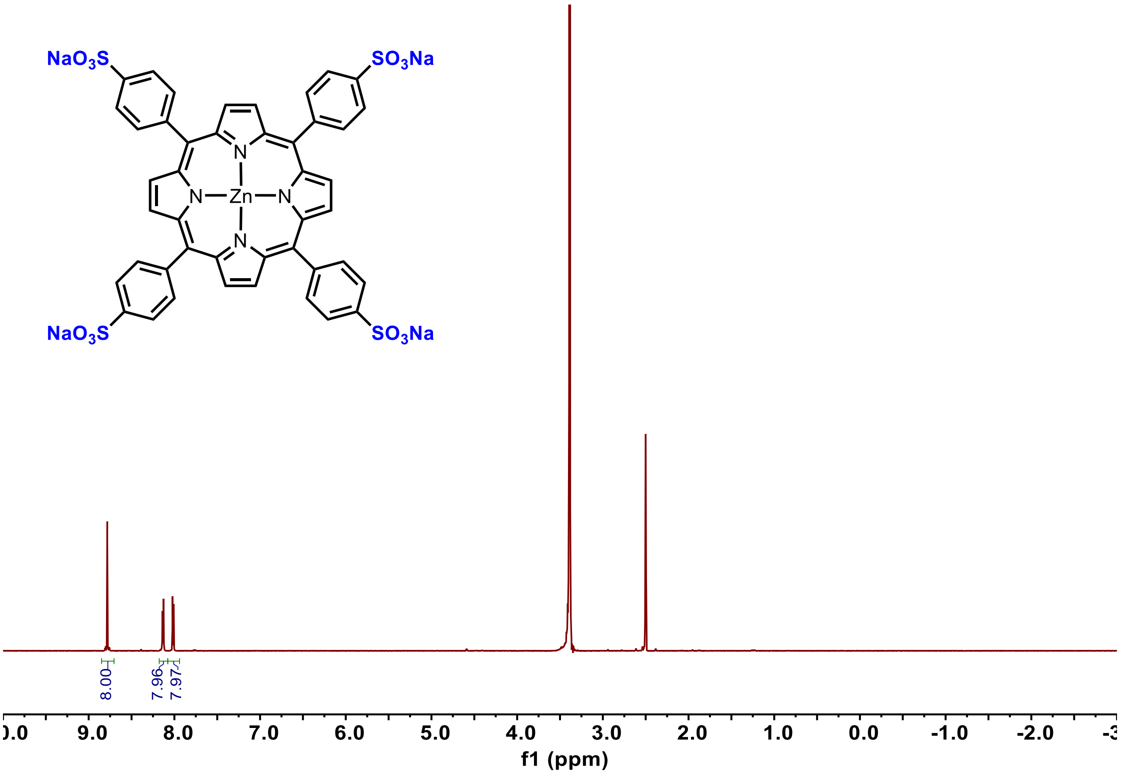

Figure S37. ^{1}H NMR (600 MHz, DMSO-d_6) of zinc(II) *meso*-tetra (4-sulfonatophenyl) porphyrin tetrasodium

5. Reference

(1) Wang, S. Y.; Li, L.; Liu, Q. H.; Urban, M. W. Self-Healable Acrylic-Based Covalently Adaptable Networks. *Macromolecules* **2022**, *55* (11), 4703-4709.

(2) Olsson, S.; Dahlstrand, C.; Gogoll, A. Design of oxophilic metalloporphyrins: an experimental and DFT study of methanol binding. *Dalton Trans.* **2018**, *47* (33), 11572-11585.

(3) Chakraborty, R.; Sahoo, S.; Halder, N.; Rath, H.; Chattopadhyay, K. Conformational-Switch Based Strategy Triggered by [18] π Heteroannulenes toward Reduction of Alpha Synuclein Oligomer Toxicity. *ACS Chem. Neurosci.* **2019**, *10* (1), 573-587.
